# Stem cells in *Nanomia bijuga* (Siphonophora), a colonial animal with localized growth zones

**DOI:** 10.1186/s13227-015-0018-2

**Published:** 2015-05-27

**Authors:** Stefan Siebert, Freya E. Goetz, Samuel H. Church, Pathikrit Bhattacharyya, Felipe Zapata, Steven H.D. Haddock, Casey W. Dunn

**Affiliations:** Department of Ecology and Evolutionary Biology, Brown University, 80 Waterman St. Box GW, Providence, RI 02912 USA; Department of Invertebrate Zoology, National Museum of Natural History, Smithsonian Institution, Washington, District of Columbia, 20004 Washington USA; Monterey Bay Aquarium Research Institute, Moss Landing, CA 95039 USA

**Keywords:** Siphonophora, *Nanomia bijuga*, Growth zone, Interstitial stem cell, i-cell

## Abstract

**Background:**

Siphonophores (Hydrozoa) have unparalleled colony-level complexity, precision of colony organization, and functional specialization between zooids (i.e., the units that make up colonies). Previous work has shown that, unlike other colonial animals, most growth in siphonophores is restricted to one or two well-defined growth zones that are the sites of both elongation and zooid budding. It remained unknown, however, how this unique colony growth and development is realized at the cellular level.

**Results:**

To understand the colony-level growth and development of siphonophores at the cellular level, we characterize the distribution of proliferating cells and interstitial stem cells (i-cells) in the siphonophore *Nanomia bijuga*. Within the colony, we find evidence that i-cells are present at the tip of the horn, the structure within the growth zone that gives rise to new zooids. Co-localized gene expression of *vasa-1*, *pl10*, *piwi*, *nanos-1*, and *nanos-2* suggests that i-cells persist in the youngest zooid buds and that i-cells become progressively restricted to specific regions within the zooids until they are mostly absent from the oldest zooids. The examined genes remain expressed in gametogenic regions. No evidence for i-cells is found in the stem between maturing zooids. Domains of high cell proliferation include regions where the examined genes are expressed, but also include some areas in which the examined genes were not expressed such as the stem within the growth zones. Cell proliferation in regions devoid of *vasa-1*, *pl10*, *piwi*, *nanos-1*, and *nanos-2* expression indicates the presence of mitotically active epithelial cell lineages and, potentially, progenitor cell populations.

**Conclusions:**

We provide the first evidence for i-cells in a siphonophore. Our findings suggest maintenance of i-cell populations at the sites of growth zones and that these sites are the main source of i-cells. This restriction of stem cells to particular regions in the colony, in combination with localized budding and spatial patterning during pro-bud subdivision, may play a major role in facilitating the precision of siphonophore growth. Spatially restricted maintenance of i-cells in mature zooids and absence of i-cells along the stem may explain the reduced developmental plasticity in older parts of the colony.

**Electronic supplementary material:**

The online version of this article (doi:10.1186/s13227-015-0018-2) contains supplementary material, which is available to authorized users.

## Background

Colonial animals provide a unique opportunity to investigate general questions about the evolution of development and to better understand development beyond embryogenesis [1–3]. Animal colonies arise when asexual reproduction is not followed by physical separation [4]. This results in many genetically identical multicellular bodies, known as zooids that are attached and physiologically integrated. Colonial species are found in many animal clades, including ascidians, bryozoans, and many cnidarians [3]. The life cycles of colonial animals require multiple developmental processes—the embryological development of the zooid that founds the colony, the asexual development of subsequent zooids, and the colony-level development that regulates larger-scale colony formation including zooid placement [3].

**Fig. 1:**
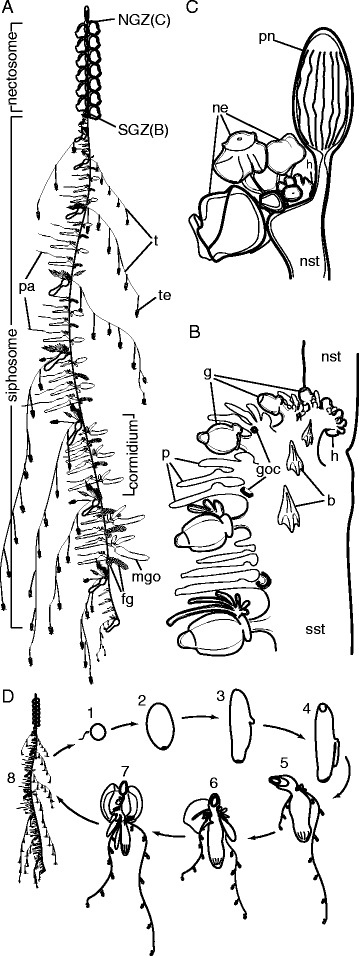
Schematic of *Nanomia bijuga*. Anterior [58] is toward the *top* of the illustrations. **a** Colony stage of the life cycle. For clarity reasons, protective bracts were not pictured and gonodendra of only one sex are shown per palpon in older parts of the colony. Approximate length of the illustrated colony was 15 cm. The side of zooid attachment within the siphosome is defined as the ventral side of the stem [58]. **b** Siphosomal growth zone and anterior part of the siphosome. Sites of gonodendra formation (goc) are located at the bases of young palpons (shown here only for the most posterior palpon in each cormidium). Gonodendra mature in older cormidia further to the posterior (**a**). **c** Nectosomal growth zone with the gas filled floating organ, the pneumatophore, at the *top*. **d** Life cycle of *Nanomia bijuga. 1.* Egg and sperm. *2.* 1.5-day-old planula. *3.* 2-day-old planula with larval tentacle bud. *4.* 2.5-day-old planula with forming pneumatophore and developing larval tentacle. The mouth opening of the protozooid is at the bottom. *5.* 1-week-old siphonula with pneumatophore and two larval tentacles bearing larval tentilla. *6.* 20-day-old siphonula with larval bract, and zooids developing on the ventral side of the protozooid. *7.* Young colony with first functional nectophore and zooids present along the elongating body of the protozooid. The elongating body of the protozooid corresponds to the future stem of the polygastric stage. *8.* Mature colony—polygastric stage with multiple gastrozooids. Original figure was adapted from [34]. *b* bract, *fg* female gonodendron, *g* gastrozooid, *goc* gonodendral i-cell cluster, *h* horn, *mgo* male gonophore, *ne* young nectophores, *NGZ* nectosomal growth zone, *nst* nectosomal stem, *p* palpon, *pa* palpacle, *pn* pneumatophore, *SGZ* siphosomal growth zone, *sst* siphosomal stem, *t* tentacle, *te* tentillum. **a–c** Modified from [59]. **d** Modified from [60].

Among colonial animals, siphonophores have both the highest degree of functional specialization between zooids and the most precise and complex colony-level organization [3]. In contrast to their benthic relatives, siphonophores have acquired a pelagic lifestyle and their zooids are arranged in very intricate repeating patterns along a linear stem (Fig. 1). Each siphonophore colony has one or two main growth zones (depending on the species) where stem elongation takes place, and new zooids arise by budding [5]. The localization of budding to such restricted zones and the consistency of budding within these zones results in very precise colony-level organization; in contrast to most other colonial animals, the zooids of a siphonophore are arranged in highly regular patterns that are consistent between colonies of the same species. This budding process has been described at a gross scale for several species [6–8]. A pro-bud that forms within the siphosomal growth zone splits into several buds that will grow into the different zooids of organized repetitive groups, the cormidia [8]. This process has been described as pro-bud subdivision [8]. The youngest zooids are closest to the growth zone and the oldest are furthest from it, providing complete ontogenetic sequences of zooid development within a colony. This greatly facilitates developmental studies. Nothing is known, however, about the cellular dynamics of colony growth. It is not known which regions have actively dividing cells, and the distributions of stem cells have never been described in siphonophores. This means that their potential role in zooid budding and colony elongation remain unknown.

Stem cells were first described in hydrozoans [9] where they are referred to as interstitial stem cells (i-cells) since they are located within interstices between epithelial cells. I-cells have not been observed in other cnidarian clades [10, 11]. Siphonophora is a monophyletic clade deeply nested within Hydrozoa [12]. Among colonial hydrozoans, i-cells have been studied in the greatest detail in *Hydractinia echinata* and *Clytia hemisphaerica* [13–15]. In *Hydractinia*, they give rise to all cell types (including epithelial cells). These i-cells are found throughout the colony and facilitate growth at different sites [2, 13], depending on environmental conditions. Hydrozoan i-cells have a distinct round or spindle shape, a high nuclear-cytoplasmic ratio, and chromatin that is less dense than that of other cells [16], which makes them conspicuous in micrographs. They also have characteristic gene expression profiles [2, 15, 17–19]. Since siphonophores mostly add new zooids within well-defined locations unlike most other colonial hydrozoans, it is important to know if stem cells are also restricted to particular regions or widely distributed as in these other species. Spatial restriction of stem cells could have a mechanistic role in restricting zooid addition in siphonophores, enabling their precise and complex growth.

Here we describe the expression of *vasa-1*, *pl10*, *piwi*, *nanos-1*, and *nanos-2* in colonies of the siphonophore *Nanomia bijuga* (Fig. 1). These genes have been frequently used to identify i-cells in other hydrozoans [17–19]. Besides expression in our target cells, several studies have found expression of the examined genes in differentiating progenitor cells and in somatic cells (e.g., [15, 18, 20, 21]). In addition, genes of the *piwi*, *vasa*, and *nanos* set have been found to be expressed in primordial germ cells and cells of the germ line across Bilateria and also within hydrozoans [15, 21, 22]. Therefore, not all cells with expression will have i-cell properties, which impacts the interpretation of our in situ hybridization results. We complement our expression data with histological studies. Our observations allow for first insights into i-cell distribution. In addition, we identify regions of cell proliferation. Our findings allow us to answer fundamental questions about colony-level development in siphonophores.

## Methods

### Collection of *Nanomia bijuga* specimens

*Nanomia bijuga* specimens were collected from the floating dock in front of Friday Harbor Labs (FHL), San Juan Island, WA (12–19 June 2011), and in Monterey Bay, CA, and adjacent waters. Monterey Bay specimens were collected on 29 Sep 2012 via blue-water diving from a depth of 10–20 m and on 28 Sep to 03 Oct 2012 by remotely operated vehicle (ROV) Doc Ricketts (R/V Western Flyer) at depths ranging from 348–465 m. Species identity between Friday Harbor and Monterey Bay specimens was established based on morphological characters. ROV-collected samples were more sexually mature compared to Monterey Bay blue-water specimens and Friday Harbor specimens and had well-developed gonodendra. After collection, specimens were kept in filtered seawater (FSW) overnight at 8 °C in the dark. Specimens for EdU labeling were collected on 19 Mar 2014 in Monterey Bay by ROV Ventana (R/V Rachel Carson) at depth ranging from 154–377 m and on 23 May 2014 by ROV Doc Ricketts (R/V Western Flyer) at a depth of 300 m. No ethical approval was needed as *Nanomia bijuga* is not subject to any animal care regulations.

### Identification and amplification of *vasa-1, pl10, piwi*, *nanos-1*, and *nanos-2* genes

We used tblastx to identify *Nanomia bijuga* homologs for *vasa-1, pl10, piwi*, *nanos-1*, and *nanos-2* in a *Nanomia bijuga* transcriptome reference using available sequence information from *Clytia hemisphaerica*, *Podocoryna carnea*, and *Hydra vulgaris*. Sequences for the *Nanomia bijuga* orthologs have been submitted to GenBank (Accession Nos. KF790888–790893). The transcriptome reference was in parts based on raw reads available at the NCBI Short Read Archive, Accession No. SRR871527 [23]. The source specimen for this library was collected on a blue-water dive in Monterey Bay on 7 Oct 2010.

### Sequence alignments and phylogenetic analysis

For each gene, a subset of significant RefSeq blast hits that matched the sampling in Kerner et al. [24] was used for phylogenetic analyses (Additional file 1). We used MUSCLE v3.8.31 [25] to generate multiple sequence alignments for each gene separately, except for PL10 and vasa that were combined into a single matrix because they are sister gene families [24]. RAxML v7.5.7 [26] was used for phylogenetic analysis with the WAG model of amino acid substitution and the Г model of rate heterogeneity. We used the non-parametric bootstrap [27] with 500 replicates for each matrix to assess support on each gene tree. The source code for the phylogenetic analyses, as well as the input fasta sequence files for all considered sequences and the output trees in newick format, are available as a git repository at https://bitbucket.org/caseywdunn/siebert_etal. Complete program settings can be found within these files.

### Whole-mount RNA in situ hybridization

**Fig. 2:**
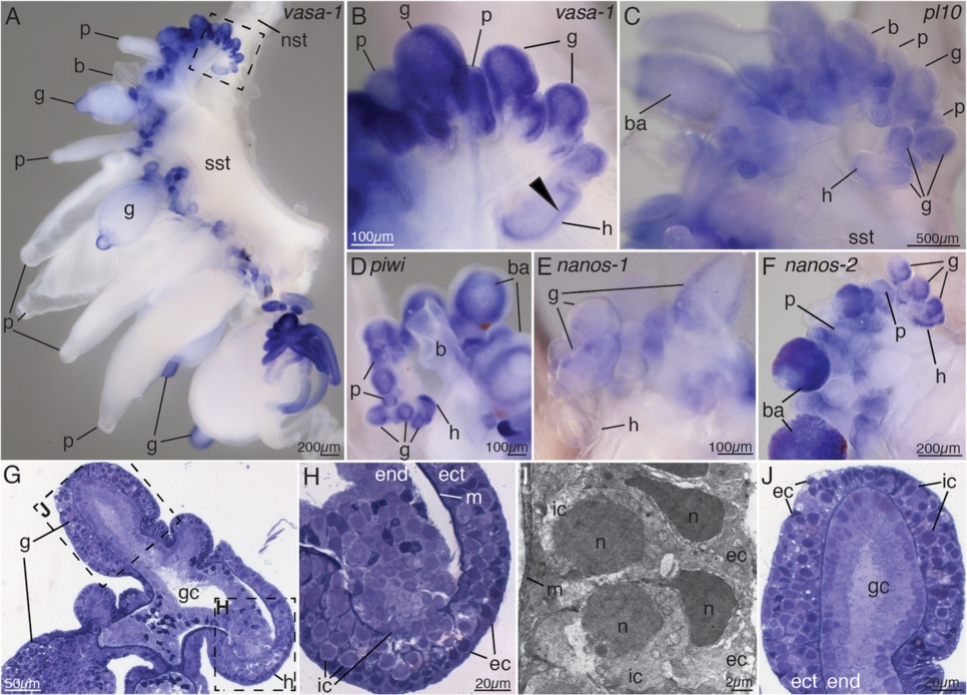
Co-localized *vasa-1*, *pl10*, *piwi*, *nanos-1*, and *nanos-2* expression and histology indicate presence of i-cells in the siphosomal growth zone. **a** Anterior part of the siphosome, stained blue for *vasa-1* transcript. Lateral view. Anterior is *up*, ventral to the *left*. **b** Close-up of growth zone region boxed region in **a**. *vasa-1* expression within the horn of the growth zone (**h**, *filled arrowhead*). **c–f** Anterior part of the siphosome, stained blue for *pl-10* (**c**), *piwi* (**d**), *nanos-1* (**e**), and *nanos-2 transcript* (**f**). **g** Semi-thin longitudinal section of the tip of the siphosomal growth zone stained with toluidine blue. **h** Siphosomal horn, close-up of box in **g. i** Transmission electron micrograph of the ectoderm of the siphosomal horn. Cells with i-cell morphology reside in between epithelial muscle cells of the ectoderm. **j** Tip of youngest gastrozooid, close-up of box in **g**. *b* bract, *ec* epithelial cell, *ect* ectoderm, *end* endoderm, *g* gastrozooid, *gc* gastric cavity, *h* horn of the growth zone, *ic* interstitial cell, *m* mesoglea, *n* nucleus, *nst* nectosomal stem, *p* palpon, *sst* siphosomal stem

**Fig. 3:**
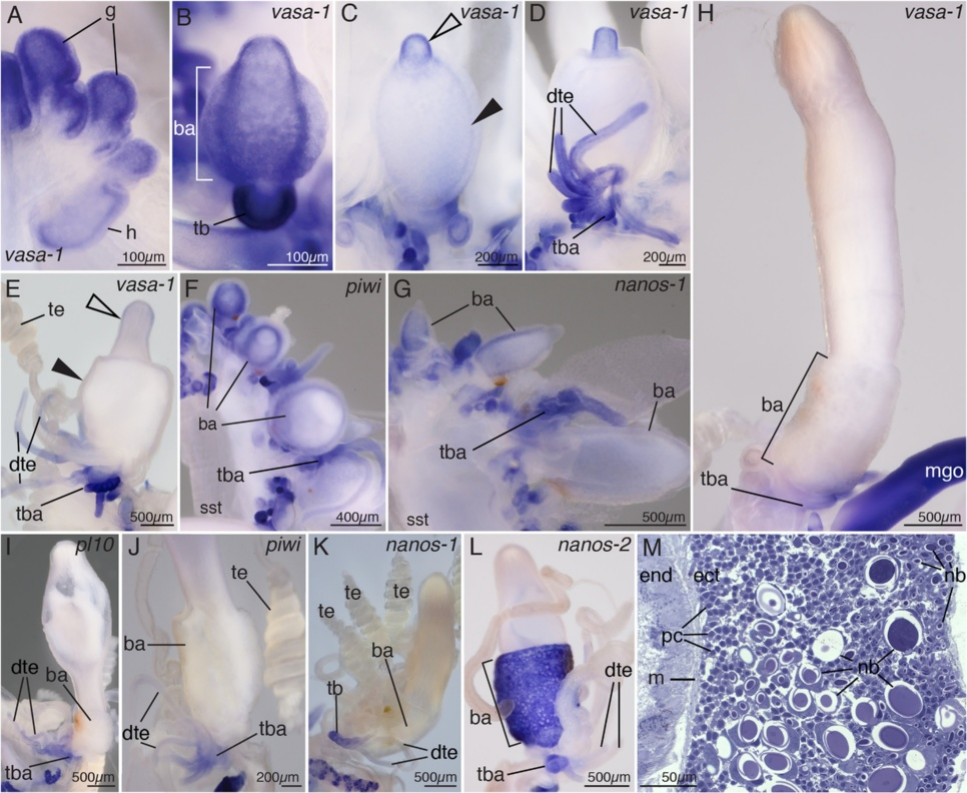
Spatial restriction of *vasa-1*, *pl10*, *piwi*, *nanos-1*, and *nanos-2* expression during gastrozooid ontogenesis indicates cell differentiation and restriction of i-cells to particular domains. **a–e**, **h** Ontogenetic series of gastrozooids, stained *blue* for *vasa-1* transcript. Distal is *up*. **a** Young gastrozooid buds close to the siphosomal horn. **b** Young gastrozooid with strong *vasa-1* expression in the developing tentacle bud. Within the basigaster region transcript was found predominantly in deeper tissue layers. Anterior view. **c** Slightly older gastrozooid with *vasa-1* expression in the gastrozooid tip (*open arrowhead*) and faint signal in deeper layers of the basigaster (*filled arrowhead*). Posterior view. **d** Early stage of tentacle formation with developing tentilla branching off the tentacle. Anterior view. **e** *vasa-1* transcript disappears from maturing gastrozooid within the developing tip (*empty arrowhead*) and from the basigaster region (*filled arrowhead*) but remains present in tentacle bases and developing tentilla. Lateral view, anterior to the *left*. **f**, **g** Ontogenetic series of gastrozooids, stained *blue* for *piwi* (**f**) and *nanos-1* (**g**) transcript. Transcript gets restricted to deeper tissue layers within the basigaster. **h–l** Mature gastrozooids stained *blue* for *vasa-1* (**h**), *pl10* (**i**), *piwi* (**j**), and *nanos-1* transcript (**k**). Expression is not detectable in the body of the mature gastrozooid. **l** *nanos-2* expression in the basigaster region and the tentacle base of a gastrozooid. **m** Semi-thin longitudinal section of a mature gastrozooid basigaster, stained with toluidine blue. Undifferentiated cells (pc) are present along the mesoglea in ectodermal tissue and nematoblasts (nb) in outer ectodermal layers. *ba* basigaster, *dte* developing tentilla, *ect* ectoderm, *end* endoderm, *g* gastrozooid, *h* horn, *m* mesoglea, *mgo* male gonophore, *nb* nematoblast, *pc* putative nematocyte progenitor cells, *tb* tentacle bud, *tba* tentacle base, *te* tentillum

**Fig. 4:**
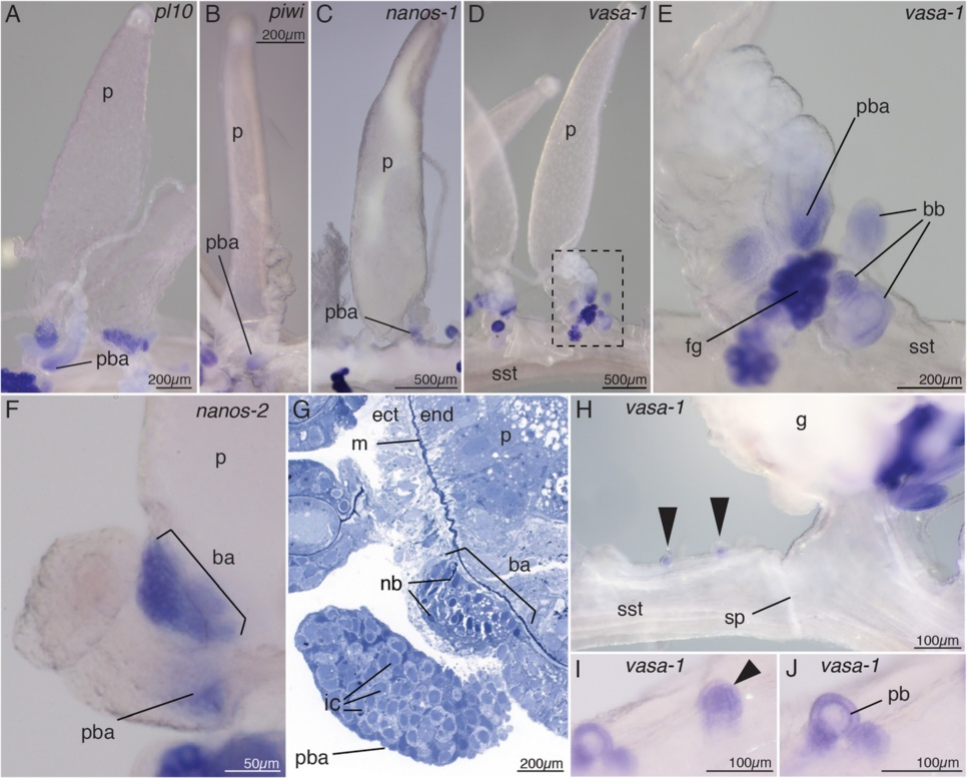
Co-localized *vasa-1*, *pl10*, *piwi*, *nanos-1*, and *nanos-2* expression in palpons suggests absence of stem cells in mature bodies and spatial restricted pools of i-cells. **a–f** Gene expression in mature palpons is restricted to the palpacle base and structures at the base of the zooid. Palpons were stained for *pl10* (**a**), *piwi* (**b**), *nanos-1* (**c**), *vasa-1* (**d–e**), and *nanos-2* (**f**). **e** Close-up of the boxed region in **d**. *vasa-1* expression is restricted to the proximal end of the palpacle base, developing bracts, and young female gonophores. **f** *nanos-2* transcript in the basigaster region and the palpacle base. Anterior is to the *left*. **g** Semi-thin longitudinal section of the palpon base, stained with toluidine blue, reveals interstitial cells in the palpacle base and nematoblasts (*nb*) in the basigaster region. Anterior is to the *left*. **h** Palpon buds at the anterior end of a cormidium (*black arrows*) with *vasa-1* expression. The sphincter region marks the posterior end of the preceding cormidium. At the site of a sphincter the hollow stem can be constricted. Anterior is to the *right*. **i** Close-up of an early palpon cluster bud (*arrowhead*). **j** Close-up of a later developmental stage of a palpon cluster with the palpon bud visible in the center and further buds laterally. *ba* basigaster, *bb* bract bud, *ect* ectoderm, *end* endoderm, *fg* female gonodendron, *ic* interstitial cell, *m* mesoglea, *nb* nematoblast, *p* palpon, *pb* palpon bud, *pba* palpacle base, *sp* sphincter, *sst* siphosomal stem

**Fig. 5:**
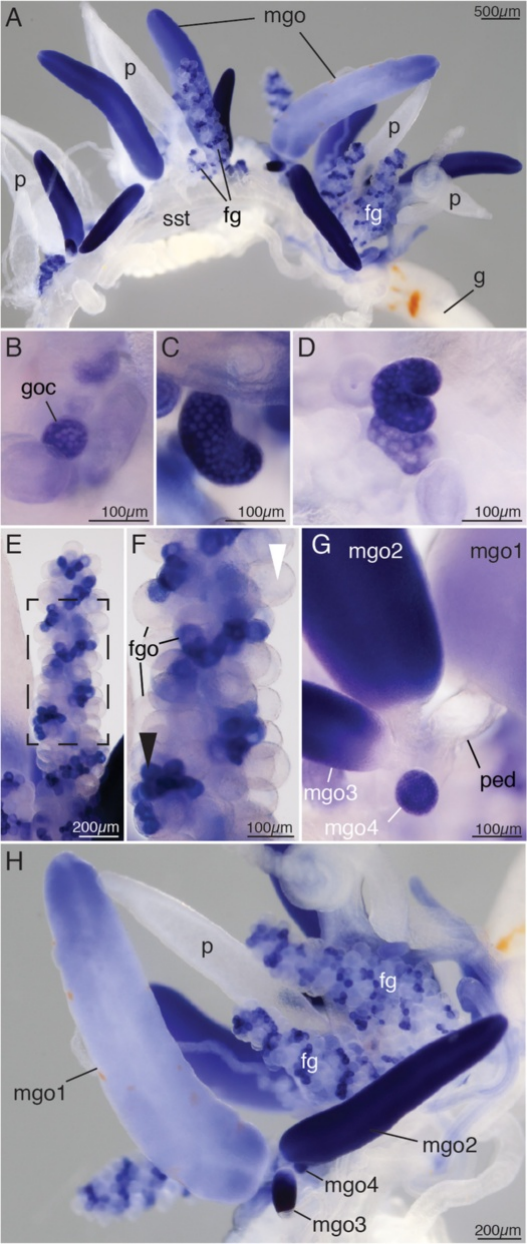
Co-expression of examined genes suggests i-cell function during gonophore formation and transient expression in the germ line, exemplary presentation of *vasa-1* transcript localization. **a** Mature cormidium, including male and female gonodendra. Anterior is to the *left*, ventral to the *top*. **b–e** Ontogenetic series of developing female gonodendra. **b** Cell cluster with *vasa-1* expression at the site of gonodendron formation at the base of a palpon. **c** Developing bean-shaped female gonodendron. **d** Developing female gonodendron starting to spiral. **e** Mature female gonodendron with developing gonophores with *vasa-1* expression and mature gonophores with *vasa-1* expression absent. **f** Close-up of female gonodendra (*boxed area* in **e**) with developing (*black arrowhead*) and mature gonophores (*white arrowhead*). Distal is *up*. **g** Close-up of the base of a male gonodendron. Later gonophores bud off the peduncle of the primary gonophore. The primary male gonophore (mgo1) is visible to the right. **h** Male gonodendron with an ontogenetic series of male gonophores, labeled mgo1-4 from oldest to youngest. *vasa-1* signal intensity decreases as the male gonophore matures. *fg* female gonodendron, *fgo* female gonophore, *g* gastrozooid, *goc* gonodendron cell cluster, *mgo* male gonophore, *mgo1* oldest male gonophore, *p* palpon, *ped* peduncle, *sst* siphosomal stem

**Fig. 6:**
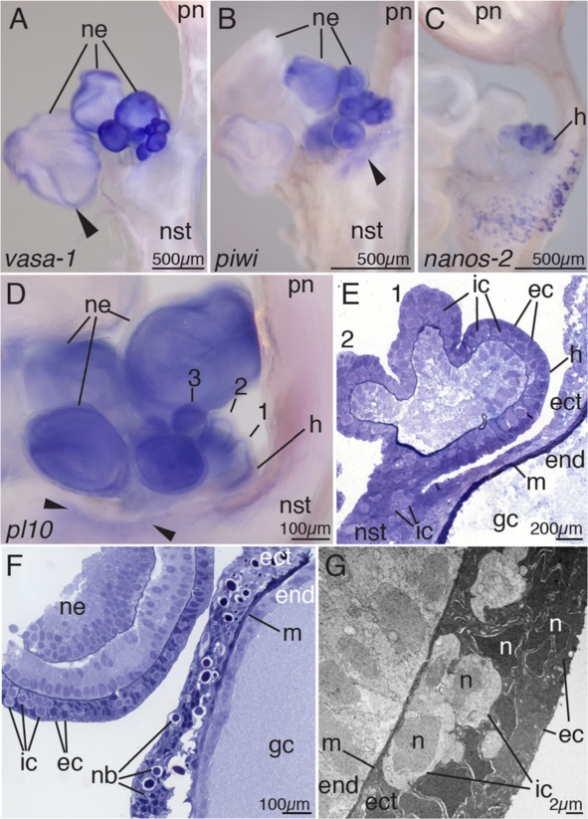
Co-localized *vasa-1*, *pl10*, *piwi*, *nanos-1*, and *nanos-2* expression and histology suggest presence of i-cells in the nectosomal growth zone. Anterior is *up* in all figures. **a** *vasa-1* transcript. Transcription was longest detectable along the nectophore ridges (*arrowhead*). **b** *piwi* expression was observed within the protruding nectosomal bulge (*arrowhead*), young buds, and developing nectophores. **c** *nanos-2* expression in the nectosomal horn and young developing buds. Signal on the nectosomal stem indicates sites of nematogenesis. **d** *pl10* transcript present in the nectosomal horn, youngest buds (1–3), young developing nectophores, and within the protruding nectosomal bulge (*arrowheads*). *pl10* expression within the horn and young buds appeared strongest in deeper layers. **e** Semi-thin longitudinal section of early nectophore buds and the horn, stained with toluidine blue. Cells with i-cell morphology could be identified in the protruding bulge of the nectosomal stem, the horn and young developing buds (1–2). **f** Semi-thin longitudinal section in the region of the nectosomal horn showing nematogenesis in the ectoderm of the nectosomal stem subtending the growth zone and interstitial cells in the ectoderm of a developing young nectophore. **g** Transmission electron micrograph showing interstitial cells in the interstices of the epithelial muscle cells within the ectoderm of a young nectophore. *ec* epithelial cell, *ect* ectoderm, *end* endoderm, *gc* gastric cavity, *h* horn of the growth zone, *ic* interstitial cell, *m* mesoglea, *n* nucleus, *nb* nematoblast, *ne* nectophore, *nst* nectosomal stem, *pn* pneumatophore

In situ hybridization was performed on Friday Harbor specimens, Monterey Bay blue-water specimens, and ROV-collected specimens and yielded consistent expression patterns. Four ROV-collected colonies per gene in four independent rounds of in situ hybridization were analyzed in detail (Figs. 2, 3, 4, 5, and 6, Additional files 2, 3, 4, 5, and 6). ROV specimens are presented in the figures since their gonodendra were more mature. Specimens were transferred into a Petri dish coated with Sylgard 184 (Dow Corning Corporation) and relaxed by adding isotonic 7.5 % MgCl_2_·6H_2_O in Milli-Q water at a ratio of approximately 1/3 MgCl_2_ and 2/3 FSW. After pinning them out in a stretched position using insect pins (Austerlitz Insect Pins, 0.2mm, Fine science tools), they were fixed in 0.5 % glutaraldehyde/4 % paraformaldehyde (PFA) in FSW for 2 min and incubated in 4 % PFA in FSW overnight at 4 °C. Mature nectophores and bracts tend to get detached when handling specimens in the dish and were therefore not accessible for analysis in all cases. Specimens were then washed three times in PTw (phosphate buffer saline and 0.1 % Tween). Dehydration was performed using EtOH with 15-min washes in 25 % EtOH/PTw, 50 % EtOH/PTw, 75 % EtOH/Milli-Q water, 2 × 100 % EtOH and then transferred to MetOH and stored at −20 °C. Use of EtOH for dehydration was empirically found to minimize tissue sloughing, detachment of endoderm from ectoderm.

Dig-labeled probes were generated using Megascript T7/SP6 kits (Life Technologies). Probe lengths in base pairs were as follows: *vasa-1*; 1,381, *pl10*; 1,233, *piwi*; 1,389, *nanos-1*; 800; and *nanos-2*; 954. Primer used for probe generation were as follows: *vasa-1_F* TTC CGG ACT ATT GCT CAA GG, *vasa-1_R* GAT CCC AGC CAT CAT CATT C; *pl10*_F ACT GCT GCA TTT TTG GTT CC, *pl10_R* TGC CTG TTG CTG GTT GTA TG; *piwi_F* CAT GCT GTG TGC TGA TGT TG, *piwi_R* GCA AAG GCC TCT TTG AAT TG; *nanos-1_F* GAA CAC TCG CTA GTT GCT GTG, *nanos-1_R* TCT ATC GGT TTT AAC TTT TGG TG; *nanos-2_F* AGT AGT GGG AGC AGC CAA TG, and *nanos-2_R* AAC CGT TGG TGG ATT GAT TC. Working concentration of mRNA probes were 1 ng/ml. In situ hybridizations were performed according to the protocol described by Genikhovich and Technau [28] with few deviations. Starting at step #27, the specimens were incubated in MAB instead of PTw. The blocking buffer composition was MAB with 1 % bovine serum albumin (BSA) and 25 % sheep serum. Anti-Digoxigenin-AP, Fab fragments (Cat.No.11093274910, Roche Diagnostics) were used in 1:2000 dilution in blocking buffer. After antibody binding, the specimens were washed in MAB instead of PBT. Once the NBT/BCIP development was stopped with water, the samples were stored overnight in 100 % ethanol followed by storage in PBS. Samples were stable in PBS for many weeks provided that the medium was exchanged regularly to prevent bacterial growth. Photodocumentation was performed using Canon MP-E 65mm Macro lens or using stereomicroscope Leica S8APO. In Fig. 2a and Additional file 2C, a stacking strategy (focal montaging) was applied to increase depth of field. Four photographs with different focal planes were merged using function “auto blend layers” in Adobe Photoshop CS 5.5. After all photo documentation was completed, specimens were stored in 4 % PFA/PBS, and the integrity of the signal has remained stable. This is a preferable long-term storage because the tissue structure is preserved. Large specimens were difficult to mount because of the size of the tissue fragments. When trying to permanently mount tissue in Euparal (BioQuip Products, Inc), the mounting procedure caused tissue damage and over time, strong unspecific staining occurred despite several washes in water after stopping the staining reaction.

### Thick and ultrathin sectioning for transmission electron microscopy

Specimens fixed as described above were washed with PBS five times for 15 min each and afterwards stored at 4 °C in the presence of sodium azide ([1 ng/ml]). Specimens were postfixed in 2 % glutaraldehyde, 4 % paraformaldehyde, 100 mM sucrose, and 100 mM sodium cacodylate buffer (SCB) overnight at room temperature. After three washes in 100 mM sucrose and 100 mM SCB for 15 min each, samples were postfixed in 1 % OsO_4_, 100 mM sucrose, 100 mM SCB overnight at room temperature. Tissue was processed for resin embedding according to the manufacturer’s instructions (Low viscosity embedding Kit, Cat. 14300, Electron Microscopy Sciences). All washes and incubations were conducted at slow agitation on a rocker table. Thick sections (0.5–0.750 μm) were prepared using glass knives, dried and counterstained for 30 s in toluidine blue (0.1 %) in sodium borate (1 %) buffer. Ultrathin sections were prepared using a diamond knife. Transmission electron microscopy (TEM) images were acquired on a Phillips 410 Transmission Electron microscope. A representative set of thick sections was deposited at the Museum of Comparative Zoology, Harvard University (catalog numbers IZ50112-50113).

### Click-iT cell proliferation assay

**Fig. 7:**
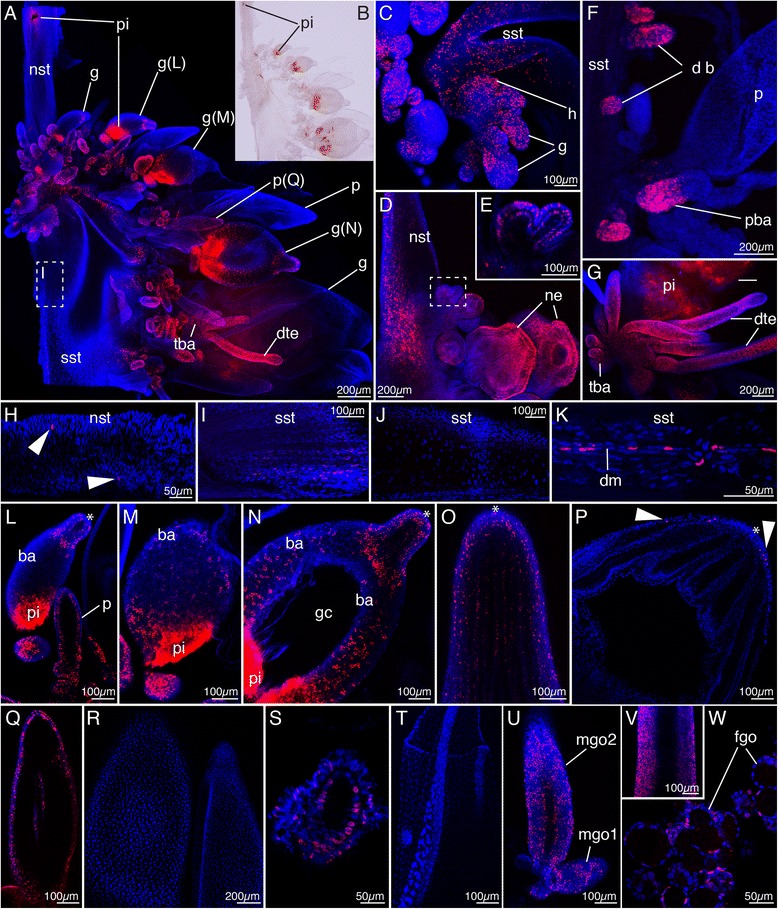
Assessment of cell proliferation in a mature colony after a five-hour EdU pulse. Nuclei of cells that divided during this interval appear *magenta*, other nuclei appear *blue*. **a** Siphosomal growth zone. **b** Bright field image of tissue shown in **a**. Pigment spots cause a red fluorescent signal. **c** Close-up of region in **a** showing EdU-labeled nuclei in the horn, young buds, and stem tissue. **d** Nectosomal growth zone with high densities of EdU-labeled nuclei along the nectosomal stem, in zooid buds and in developing nectophores. **e** Horn to the *left* and youngest bud, close-up of box in **d. f** Siphosomal stem fragment (SF2) with young developing buds, mature palpon, and palpacle base. **g** Tentacle base and developing tentilla at the base of the gastrozooid basigaster. **h** Posterior part of the nectosomal stem of the colony shown in **d**. EdU-labeled nuclei are sparsely scattered (*arrowheads*). **i** EdU-labeled nuclei along the stem posterior to the siphosomal growth zone, close-up of box in **a. j** Posterior siphosomal stem fragment (SF2) with EdU-labeled nuclei absent. **k** Siphosomal stem fragment (SF1) with EdU-labeled nuclei along the dorsal canal. **l–p** Ontogenetic series of gastrozooid development. **l–n** Close-ups of gastrozooids (g) shown in **a. o** Developing hypostome of a gastrozooid with high densities of EdU-labeled nuclei. **p** Hypostome of a mature gastrozooid from siphosomal fragment (SF2) with few EdU-labeled nuclei (*arrowheads*). **q** Close-up of developing palpon (p) shown in **a. r** Two mature palpons from siphosomal fragment SF2 with EdU-labeled nuclei absent. **s** Young developing bract from siphosomal fragment (SF2) with EdU-labeled nuclei. **t** Mature bract from siphosomal fragment (SF3) with EdU-labeled nuclei absent. **u** Developing male gonophores from siphosomal fragment SF2. **v** Mid-section of a late male gonophore from siphosomal fragment SF3. **w** Developing female gonophores with EdU-labeled cells. **a–c, f–g** Anterior is *up*, ventral to the *right*. **d**, **e** Anterior is *up*, dorsal to the *right*. **h–j** Anterior to the *left*, lateral view. **k** Anterior to the *left*, dorsal view. **l–w** Distal is *up. b* bract, *ba* basigaster, *db* developing bud, *dte* developing tentilla, *dm* dorsal midline, *fgo* female gonophore, *g* gastrozooid, *gc* gastric cavity, *h* horn of the growth zone, *mgo* male gonophore, *ne* nectophore, *nst* nectosomal stem, *p* palpon, *pba* palpacle base, *pi* pigment, *sst* siphosomal stem, *tba* tentacle base, * hypostome

After collection, specimens were kept at 5–7 °C overnight or up to 2 d in the dark. Each specimen was truncated to a colony length of approximately 8 cm, in relaxed state, by surgical removal of posterior parts of the siphosome. This was done to ensure comparable amounts of tissue in different incubations. Colonies (C1–C6) were incubated in 50 ml volume per individual at click-iT® EdU concentrations of 100 μM (five specimens, C1–C5) and 20 μM (one specimen, C6) in FSW for 5 h at a temperature of 5–7 °C. Both concentrations yielded comparable results. Specimens were transferred into a Petri dish coated with Sylgard 184 and fixed as described above for in situ hybridization specimens. Dehydration was performed using EtOH with 15-min washes in 25 % EtOH/PTw, 50 % EtOH/PTw, and 2 × 75 % EtOH/Milli-Q water; specimens were stored at −20 °C. To compare cell proliferation in different regions of the colony, the specimens were dissected prior to the click-iT reactions. The nectosomal and the siphosomal growth zones including adjacent stem regions and up to three siphosomal fragments (SF1, SF2, SF3) with fully-grown zooids attached to the stem were transferred into wells of a 24-well plate (Costar 3524, Corning Incorporated). Siphosomal fragments (SF1, SF2, SF3) were taken at distances of approximately 1.5, 3, and 4.5 cm in posterior direction from the siphosomal horn and included at least one mature gastrozooid. The stem length in these tissue samples varied in between 2.5 mm and 1 cm. The tissue was rehydrated and permeabilized at room temperature using 10-min washes in 50 % EtOH/PBS, 25 % EtOH/PBS, 2 × PBS, 2 × 3 % BSA in PBS, 0.5 % Triton X in PBS (20 min) and 2 × 3 % BSA in phosphate buffered saline (PBS). The click-iT reaction was performed according to the manufacturer’s instructions (Click-iT® EdU Alexa Fluor® 594 Imaging Kit, C10339, Life Technologies). Before mounting, the tissue was counterstained with DAPI (D1306, Life Technologies) solution at a concentration of 2 ng/μl. The tissue was mounted in Vectashield (H-1000, Vector laboratories) and analyzed on a Zeiss LSM 510 Meta Confocal Laser Scanning Microscope. The overview shot presented in Fig. 7a was generated manually in Adobe Photoshop CS6 by merging eight individual shots, which were taken consecutively using an identical focal plane. Comparisons between zooids of different developmental stages were made within one specimen when possible. The fixation and mounting procedure however rendered particular tissues inaccessible for confocal analyses in some cases. Photographs for presentation purposes had therefore been acquired across colonies on few occasions. Photographs shown in Fig. 7 summarize the observations made across all six analyzed specimens. A quantitative analysis of cell division was however not accessible at this time. Images were taken of the tissue of colony C1 (Fig. 7a–c, g, i, j, l–o, q, r), colony C2 (Fig. 7f, k), colony C3 (Fig. 7p, s–w), and colony C6 (Fig. 7d, e, h), respectively.

## Results

We provide the first evidence for the presence of i-cells in siphonophores and describe their distribution using whole-mount in situ hybridization and histology. Broadly sampled phylogenetic analyses indicate that we could identify *vasa-1*, *pl10*, *piwi*, *nanos-1*, and *nanos-2* orthologs in *Nanomia bijuga* (Additional file 1A–C). Negative controls with sense probes were performed for in situ hybridizations of all genes in all zooids, and none were positive. Key findings are presented in the figures of the main manuscript. All in situ results and negative controls are summarized in Additional files 2, 3, 4, 5, and 6. The whole-mount in situ hybridization techniques used in this study enabled us to identify tissue regions with co-expression of genes but lacked the spatial resolution to confirm if these genes were co-expressed in the same cells within these regions. We interpret the co-localized expression of all examined genes as a broad proxy for the presence of i-cells, though domains of expression may be supersets of domains with i-cells due to expression in other cell types, including germ cells, progenitor cells, and potentially somatic cells (e.g., [15, 18, 20, 21]). It will require follow-up studies to tie expression to particular cell types, which we have recently described in greater detail [29]. We confirmed the presence of cells with i-cell morphology in select regions that showed expression. We interpret the absence of expression of examined genes as evidence of the absence of i-cells. In some cases we find clear differences between the expression domains of different genes, which we describe in greater detail below. These differences are likely due to some expression of some genes in cell types other than i-cells, including germ cells and nematoblasts. In cases where expression appeared co-localized across all five examined genes, we only feature exemplary results for *vasa-1* in some of the main figures.

### Evidence for the presence of i-cells in the horn of the siphosomal growth zone

The siphosomal growth zone produces most zooids in *Nanomia bijuga* (Fig. 1a, b). The general structure of the *N. bijuga* siphosomal growth zone, as well as its budding process, has previously been described [7]. The zooids are arranged in repeating groups, known as cormidia. The budding sequence that produces cormidia and the zooid arrangement within them are highly organized (Fig. 1a, b, [7]). The siphosomal growth zone has a protrusion at its anterior end—the horn (labeled h in Fig. 1b). Pro-buds form at the tip of the horn and then subdivide into zooid buds as they mature and are carried to the posterior. These buds give rise to five different zooid types—gastrozooids (feeding polyps), palpons (polyps with function in circulation, defense, and digestion), bracts (defense), and female and male gonophores (gamete production) [5].

All examined genes were found to be expressed at the tip of the siphosomal horn and in all buds and young zooids within the siphosomal growth zone, with *nanos-1* showing the lowest signal (Fig. 2a–f). Semi-thin sections and TEM analysis confirmed the presence of two types of cells within the ectoderm of the siphosomal horn, epithelial cells, and undifferentiated cells with i-cell morphology (Fig. 2g–i). Within the horn, cells with i-cell morphology were also found in the endoderm (Fig. 2h). The mesoglea within the horn appeared discontinuous suggesting that there may be migratory activity of i-cells between ectoderm and endoderm (Fig. 2h). In the endoderm of young zooid buds, however, no cells with i-cell morphology were observed. Nuclei of endodermal cells were located close to the mesoglea (Fig. 2j). Both epithelial cells and cells with i-cell morphology were found in the ectoderm of young zooids (Fig. 2j).

### Co-localized *vasa-1*, *pl10*, *piwi*, *nanos-1*, and *nanos-2* expression suggests spatial restriction of i-cells during zooid development

The distal portion of the pro-bud gives rise to the gastrozooid—the feeding zooid (Figs. 1b, 2b, and 3). Young gastrozooid buds had expression of all genes (Figs. 2b–f and 3a). The basigaster, a specialized region of nematocyst formation in siphonophores [5], was evident in young gastrozooid buds as a thickening of the proximal ectoderm (Fig. 3b). In the course of basigaster development, expression of all examined genes, except *nanos-2* (Fig. 3l), became restricted to deep basigaster ectoderm (Fig. 3b–g, Additional file 3C, D) and then decreased until a signal was no longer detectable in mature gastrozooids (Fig. 3h–k, Additional file 4F). *nanos-2* expression persisted in the basigaster region of gastrozooids of all ontogenetic stages (Figs. 2f and 3l, Additional file 6C, E, G, H). This finding was consistent with previous studies that indicated a *nanos-2* function in nematocyst formation [15, 30]. Within the basigaster, *nanos-2* seemed to be co-localized to the same region as minicollagen (see [31]), which is known to be involved in capsule formation [32]. Though *vasa-1, pl10*, *piwi*, and *nanos-1* transcripts were not detected in basigasters of mature gastrozooids (Fig. 3h–k), undifferentiated cells were still found along the mesoglea (Fig. 3m) indicating the presence of a determined progenitor cell population which gives rise to nematocytes but has lost interstitial stem cell transcriptional signatures. Immature nematocysts were observed in the outer layers of the mature basigaster (Fig. 3m). The gene *vasa-1* was expressed in the same regions of the young gastrozooids as *pl10*, *piwi*, and *nanos-1*. In addition, it was expressed in both the ectoderm and endoderm of the tips of young gastrozooids (Figs. 2a and 3b–e).

Each gastrozooid has a single tentacle attached at its base. The tentacle has side branches, known as tentilla, which bear packages of nematocysts at their termini (Fig. 1a, [5]). All examined genes were expressed in the tentacle bases throughout all ontogenetic stages of gastrozooids (Fig. 3b, d–g, h–l). The expression domains, however, differed between genes. Whereas *nanos-2* expression was restricted to the very proximal end of the tentacle and very early tentilla buds (Fig. 3l, Additional file 6H, I), signal for the other four genes persisted in developing tentilla as well (Fig. 3d, e, j, k). None of the examined genes were expressed in mature tentilla (e.g., in Fig. 3j, k).

Anterior to each gastrozooid, a series of buds develop into palpons—zooids thought to have a function in circulation of gastrovascular content, digestion, and defense (Fig. 1b, [33]). Like gastrozooids, each palpon has a single tentacle (Fig. 1a), which is known as a palpacle [5]. The palpacle is, in contrast to the gastrozooid tentacle, unbranched and nematocysts can be found along its entire length. As in gastrozooids, strong expression was detected for all examined genes in young palpons within the growth zone, and expression disappeared from later developmental stages (e.g. Fig. 2a–d, f). Expression was absent from mature palpons (Figs. 2a and 4a–d, f), except for *nanos-2*, which remained expressed in a small domain at the proximal end of the palpon (Fig. 4f, Additional file 6J). Unlike in gastrozooids, this *nanos-2* expression domain did not extend around the entire zooid but was restricted to a small patch close to the palpacle base (Fig. 4f). Semi-thin sections indicated this patch as a site of nematogenesis (Fig. 4g), suggesting that it is equivalent to the basigaster of gastrozooids. These similarities between gastrozooids and palpons were consistent with the hypothesis that palpons are derived gastrozooids that lost the ability to feed, i.e., they lack a mouth opening [5]. Expression of all examined genes was found at the proximal end of the palpacle (Fig. 4a–f, Additional file 2G). Densely packed, undifferentiated cells with i-cell morphology were present within palpacle bases (Fig. 4g). Additional secondary palpons are added at the anterior end of mature cormidia, and gonodendra form laterally from these secondary palpons [5]. We frequently found small buds anteriorly from the youngest primary palpon, which were at the sites where these secondary structures arise. All examined genes were found to be expressed in such buds (Fig. 4h–j).

Bracts are protective zooids, which can be found laterally along the siphosomal stem but also associated with palpons and gastrozooids (Fig. 1b, [7]). They are of scale-like morphology and function as protective shields. As in gastrozooids and palpons, all examined genes were expressed in early developing bract buds (shown for *vasa-1*, Fig. 4e), but expression was absent in older bracts once the typical bract morphology became obvious (shown for *vasa-1*, Fig. 2a).

### *vasa-1*, *pl10*, *piwi*, *nanos-1* and *nanos-2* expression in sexual zooids

While some siphonophore species are dioecious, a colony of *Nanomia bijuga* produces gametes of both sexes [5]. Gametes are produced by gonophores, each of which is either male or female. These gonophores are arranged into groups called gonodendra [5], which each exclusively bear male or female gonophores. Gonodendra are attached directly to the stem and develop laterally at the base of the palpon peduncle. There are gonodendra of both sexes associated with each palpon, one male and up to two female gonodendra. The locations of these male and female gonodendra alternate between adjacent palpons (Figs. 1a and 5a, [5]).

Female gonodendron formation has been described previously [34] as follows. Female gonodendra start to form as small buds protruding at the base of the palpon peduncle. Germ cells develop in between endoderm and ectoderm. Each gonophore within the female gonodendron contains a single egg. The egg is enclosed by a thin ectodermal layer within the developing female gonophore. Two lateral canals form from endodermal epithelial cells within the gonophore. The mature gonophore is attached to the blind-ending central stalk of the gonodendron by a delicate peduncle.

In situ hybridizations for all five genes yielded identical expression patterns in gonodendra. Findings for *vasa-1* are summarized in Fig. 5 and are representative for the other four examined genes. Close to the growth zone, the first indication of gonodendron development was round clusters of cells with strong expression on the stem at the base of the young palpons (Figs. 1b and 5b, Additional file 6M). These clusters were visible before bud formation became obvious, and male and female clusters were morphologically indistinguishable from each other at this stage. In situ hybridization revealed expression of all five genes in a helical pattern in the mature female gonodendron. This pattern corresponds to a previously unobserved helical morphological organization (Fig. 5e, f, h, Additional files 3K, 4M, 5J, and 6G, P). The gonodendron buds started to twist early in development, and a stronger signal was observed on the outer side of the developing stalk away from the axis of the helix (Fig. 5c, d, Additional file 6N, O). This pattern persisted during the first turns until the gonodendron took on an appearance reminiscent of clusters of grapes. At this stage, all examined genes were strongly expressed in all gonophores along the gonodendron, and the helical organization was not apparent. Helical organization became obvious again in later ontogenetic stages (Fig. 5e, f, Additional files 3K, 4M, 5J, and 6G) when expression decreased in mature gonophores (Fig. 5e, f). The presence of signal in immature gonophores distributed in a helical pattern along the gonodendron indicated that new gonophores were produced along one side of the entire twisted stalk of the gonodendron. The chirality of the helices changed with the site of attachment. Gonodendra attached on the left side of a palpon showed a clockwise directionality of turns.

The male gonodendron starts with the formation of a primary gonophore, which is cone shaped. Secondary and tertiary gonophores bud off the delicate peduncle of the primary gonophore (Fig. 5g). The male gonophore is an elongated structure with a massive population of putative germ cells in the ectoderm (see [29]). All examined genes were strongly expressed in young and medium-sized gonophores, but signal intensity was lower or absent in gonophores close to or at maturity (Fig. 5h, Additional files 3M, 4N, 5L, and 6Q). The absence of graded signals along the proximal-distal axis suggests that sperm maturation took place along the entire gonophore.

### Nectosomal growth zone has a similar structure as the siphosomal growth zone

*Nanomia bijuga*, like most other siphonophore species, has a nectosomal growth zone (Fig. 1c) near the anterior end that produces the swimming zooids, called nectophores, which propel the whole colony through the water [5]. All examined genes were strongly expressed in the nectosomal growth zone at the tip of the horn, in nectophore buds, and in young developing nectophores (Fig. 6a–d, Additional file 5A). Co-localized expression of all genes and histological sections suggested the presence of i-cells in the thickened region of the nectosomal stem, the horn of the growth zone, and young nectophore buds (Fig. 6a–d, Additional file 5A). In case of *vasa-1*, the transcript persisted longest along the ridges of the nectophores (Fig. 6a). Older nectophores were free of gene transcripts in case of all examined genes (e.g., Fig. 6b–d, Additional file 5A). In contrast to the other four genes, *nanos-2* expression was restricted to the very youngest nectophore buds (Fig. 6c). In addition, in the stem subtending the growth zone, the transcript was detected on the nectosomal stem in a salt and pepper pattern (Fig. 6c). Sections revealed developing nematocysts in this region of the stem (Fig. 6f). Undifferentiated cells with interstitial cell morphology were identified in the ectoderm of the horn and developing nectophores (Fig. 6e–g).

### Co-localized *vasa-1*, *pl10*, *piwi*, *nanos-1* and *nanos-2* expression is found in a subset of regions with high rates of cell proliferation

A qualitative assessment of cell proliferation revealed high densities of EdU-labeled nuclei in domains with expression of all examined genes (compare Figs. 2a and 7a). Specifically, EdU-labeled nuclei were found in the horns of both growth zones as well as in young buds and developing zooids both within the growth zones and along the siphosomal stem (Fig. 7a–f). In all analyzed tissue samples, the palpacle bases consistently had strong EdU labeling in developing palpons as well as in mature palpons (Fig. 7f). Tentacle bases and developing tentilla were also strongly EdU labeled in gastrozooids (Fig. 7a, g). In addition, high densities of EdU-labeled nuclei were found in stem regions at the level and adjacent to both growth zones (Fig. 7c, d, i), where only a few or no EdU-labeled nuclei were identified in posterior regions of the nectosomal (Fig. 7h) and siphosomal (Fig. 7j) stem. These EdU-labeled regions in the stem are the main sites of stem elongation in *Nanomia bijuga*. Interestingly, these stem regions were devoid of *vasa-1*, *pl10*, *piwi*, *nanos-1*, and *nanos-2* gene expression (compare Figs. 2b and 7c and Figs. 6a, b and 7d). Conspicuous cell division was occasionally observed along the dorsal midline of the stem (Fig. 7k), whereas no signal was obtained in these regions in the in situ hybridizations. The number of EdU-labeled cells in a particular zooid type decreased with level of maturity, and in many cases, no proliferative activity was found in mature zooids (Fig. 7l–t). In developing male gonophores, our EdU assay showed a large number of dividing cells in the ectoderm (Fig. 7u, v). In developing female gonodendra, EdU-labeled nuclei were consistently detected in developing gonophore bells (Fig. 7w).

## Discussion

### Interpreting the biological implications of expression, proliferation, and cell morphology

We interpret the regions in *Nanomia bijuga* without expression of the examined genes as devoid of i-cells. We find, using histological observations, that i-cells are present in at least some of the regions with expression of examined genes, though some expression of the examined genes may be in other cell types such as progenitor cells, e.g., nematoblasts or neuroblasts. Clusters of cells that develop into gonodendra may contain interstitial stem cells, with roles in gonophore formation, and primordial germ cells. All regions with expression of the examined genes are also regions of elevated cell proliferation. Double-labeling experiments that can co-examine gene expression, cell proliferation, and morphology at the cellular level would be required to fully understand the biological implications of the gene expression patterns shown here.

### A cellular perspective on differences in growth and form between siphonophores and other hydrozoans

**Fig. 8:**
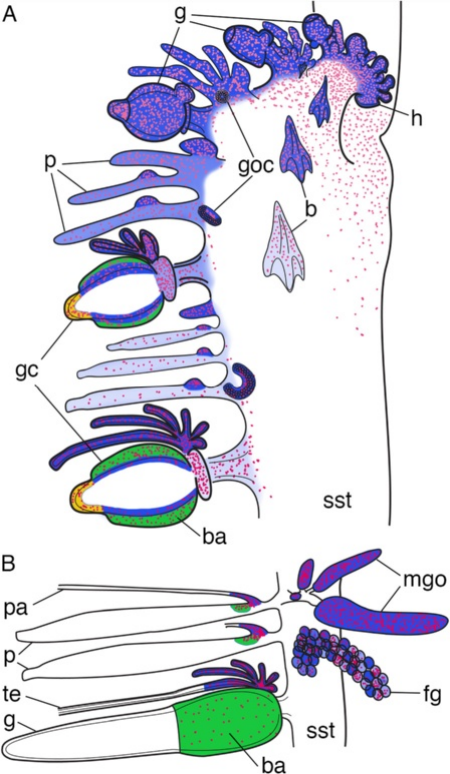
Schematic of i-cell distribution and cell proliferation in *Nanomia bijuga. Shades of blue* indicate density of i-cells, as indicated by co-localized expression of the examined genes. *Magenta dots* indicate cell proliferation. **a** Siphosomal growth zone and anterior part of the siphosome. Two gastrozooids (gc) are represented as cross sections. I-cells (*blue*) get restricted to deeper layers of the basigaster. *nanos-2* (*green*) continues to be expressed in outer layers of the basigaster, and *vasa-1* (*yellow*) continues to be expressed within the developing hypostome. **b** Older siphosomal stem fragment with a mature gastrozooid, two palpons, and developing gonozooids. Cell proliferation is spatially restricted to tentacle and palpacle bases and to developing gonophores. *b* bract, *fg* female gonodendron, *g* gastrozooid, *gc* gastrozooid cross section, *goc* gonodendral i-cell cluster, *h* horn, *mgo* male gonophore, *p* palpon, *pa* palpacle, *sst* siphosomal stem, *te* tentacle

The distribution of i-cells suggested by the histological and gene expression analyses presented here (summarized in Fig. 8) differs in several key respects from the distribution of i-cells described from other hydrozoans. These differences, along with differences in the distribution of cell proliferation, may help explain the development and evolutionary origins of the unique colony-level development and morphology of siphonophores.

Cell proliferation is high within the horn and young developing zooids of the siphonophore *Nanomia bijuga*. It then decreases in the course of zooid development and was mostly absent in mature zooids (Fig. 8). This is in contrast to benthic colonial relatives such as *Tubularia* and *Hydractinia* where mitotic activity is maintained in mature gastrozooids [35]. Cell proliferation persists at the base of tentacles and palpacles, indicating continuous growth, as well as in gamete producing zooids. We also see rates of high proliferation in the stem within the growth zones. This is the first confirmation of restricted growth in the stem.

Co-localized expression of the examined genes, in conjunction with more restricted histological observations, suggests that i-cells are present in a subset of the regions with high rates of cell proliferation. These regions include the horn and developing zooids (summarized in Fig. 8). More importantly, we could not identify in situ signals of expression of the examined genes along the stem of the colony, either within the growth zone or in the stem between mature zooids. This indicates the absence of i-cells in these regions. This has several important implications. First, even though we could not clarify the identity of these dividing cells, it suggests that epithelial cell division is responsible for stem elongation in *N. bijuga* and does not require i-cells. Second, the lack of i-cells in the stem differs from benthic colonial hydrozoans that have widely distributed i-cells along their stolons and plastic growth. In the hydrozoan *Hydractinia echinata*, i-cells reside in the stolon system, which interconnects the different bodies of the colony [13, 14]. These i-cell distribution patterns allow for addition of new zooids at various sites along the entire stolon system, and different colonies of the same species do not have the exact same organization of zooids relative to each other.

### The origin, fate, and developmental potential of siphonophore i-cells

The potency, i.e., the ability to differentiate into other cell types, of siphonophore i-cells remains unknown. The interstitial cell lineage in other hydrozoans that have been examined consists of pluripotent i-cells that give rise to unipotent progenitor cells [9, 36]. These stem cells give rise to somatic cells such as nerve cells, gland cells, nematocytes, or gametes [2, 36, 37]. Previous work has revealed diversity within Hydrozoa in the potency of i-cells. In the freshwater polyp *Hydra*, i-cells are pluripotent but cannot give rise to epithelial cells [37–39]. In contrast, the i-cells of the marine colonial hydrozoan *Hydractinia* can give rise to all cell types including epithelial cells [2, 13, 14].

In several cases, we identified differentials in the expression domains of the examined genes, which we interpret as hints for the presence of determined progenitor cells. For instance, *vasa1* continues to be expressed in the tip of the developing gastrozooid (Fig. 8), and the expressing cells may give rise to a tissue-specific yet unidentified cell type. Exclusive expression of *nanos2* indicates the presence of nematoblasts since a role of *nanos2* in nematocyst-formation pathways has been demonstrated previously (Fig. 8) [15, 30]. Migrating progenitor cells may be the mechanism by which the elongating stem gets replenished with somatic stem cells such as nerve cells, since these cell types are present within stem tissue [29, 40, 41]. Some of the mitotically active cells in the stem region may therefore be amplifying progenitor cells, which have lost i-cell specific signatures. Two giant nerve fibers run along the dorsal midline of the siphosomal stem of *N. bijuga*, which function as rapid conduction pathways and for which a syncytial character has been reported [40, 42]. Dividing cells along this dorsal midline (Fig. 7k) in older parts of the colony may point to the presence of nerve progenitor cells ultimately contributing to giant axon fibers. The cellular identity of these cells could, however, not be established in this study. Migratory activity of progenitor cells with already determined fates, e.g., nematoblasts and neuroblasts, has been frequently demonstrated in of a variety of hydrozoan species (e.g., [43–47].

The elongating stem of the siphonophore colony corresponds to the body column of the primary polyp, which is formed in embryogenesis (Fig. 1d, [5]). Our data suggest that pluripotent i-cell populations get restricted to the sites of future growth zones during development of the primary polyp in the course of growth-zone establishment. Siphonophores allow for the analysis of complete ontogenetic series of particular body types, which are arranged along the stem by age, which greatly facilitate the present study. The primary pattern we observe is that as zooids mature, the distribution of i-cells within them becomes more restricted, perhaps because they are not renewed as cells differentiate in the course of maturation.

There is one region where the pattern of expression may not be explained by depletion of i-cells in the course of differentiation—the sites where secondary palpons and gonodendra are added at the anterior end of each cormidium. These structures arise at patches with expression of the examined genes (Fig. 4h–j). It could well be that the i-cells in these patches are incorporated into the developing cormidium early in the growth zone, but the temporal resolution available in this study cannot exclude other options. Alternative explanations could involve migration of i-cells to these patches. Migration, however, would require gene expression at intermediate regions along the stem of the colony, which we did not observe. Another possible explanation is that epithelial cells could undergo transdifferentiation and give rise to i-cells in these patches. Transdifferentiation has been reported for hydrozoan relatives. For instance, in *Podocoryna carnea*, differentiated striated muscle cells have the potential to undergo pluripotent transdifferentiation under appropriate conditions [48, 49].

The restriction of i-cells to particular sites in the siphonophore colony may explain previous observations of reduced regenerative capacities in siphonophores relative to other colonial animals [50]. This reduced regenerative capacity also suggests that transdetermination or transdifferentiation events are absent or rare. This highlights the restriction of stem cell pools to budding zones, in combination with spatial patterning during pro-bud-subdivision early in the growth zone, as the key innovation, which may have lead to a reduced plasticity, enabled a far more precise, unique, and highly organized model of growth observable in siphonophores.

### The analogy of siphonophore growth zones to plant meristems

Others have likened regions of growth in hydrozoans to land plant meristems and suggested localized growth at tips of stolons or hypostomes [51, 52]. Such localized cell proliferation and meristematic character of these regions could, however, not be confirmed in later studies [53, 54]. Cell proliferation was rather found present along the entire stolon systems [53–56]. Berking et al. (2002) described a meristem-like organ in the thecate hydrozoan *Dynamena pumila*. In a *D. pumila* colony each stem has a growing tip, which, usually, neither ends in a stolon tip nor as a polyp but grows out as to form the stem [57]. This growing tip frequently splits into three primordia, two of which give rise to lateral buds, which develop into polyps, and the third forming a new growing stem tip.

Within Hydrozoa, siphonophores seem to have taken the degree of spatial restriction of a pluripotent pool of cells and proliferating cells to an extreme. This makes the plant analogy a particularly interesting one though the cellular organization in plants and hydrozoans clearly differs. Analogously to a plant meristem, which produces structures that develop into functional organs, siphonophore buds generated laterally from the horn within the growth zone develop into specialized bodies. Both the meristem in plants and the horns within the growth zones can be characterized as restricted morphogenetic fields that harbor constantly dividing cells. In both cases, new cells are produced for expansion and tissue differentiation. In the case of the siphonophore horn, cell division of endodermal and ectodermal cells as well as nested amplifying interstitial cells generate tissue available for bud formation. Cells differentiate and zooids mature as these newly formed structures are carried away from the horn. Our observations of cellular proliferation and stem cell distribution within the siphonophore colony allow for a more detailed extension of the analogy from observable patterns to the cellular dynamics that give rise to those patterns.

## Conclusions

We provide the first evidence for i-cells in siphonophores and describe general patterns of i-cell distribution as suggested by expression patterns of select genes and targeted histological examinations. These observations, in combination with cell proliferation assays, suggest a general model for cellular dynamics of colony-level growth and development in siphonophores (Fig. 8) characterized by the restriction of i-cells to particular sites. In other colonial hydrozoans such as *Hydractinia*, i-cells enable budding along the stolon system. This leads to variable growth patterns. Unlike in these other colonial hydrozoans, we did not observe i-cells along the stem of the siphonophore colony but found evidence of i-cell populations within the horns of the two growth zones. These populations appear to be the main source of i-cells in the colony. During zooid ontogenesis we find evidence for progressive spatial restriction of i-cells to domains for which continuous growth is indicated by cell proliferation, such as tentacle or palpacle bases. Stem cell differentiation and depletion in addition to spatially restricted maintenance appear to be the main mechanisms underlying i-cell distribution. Spatial restriction of i-cells may have enabled a complex and precise colony-level development at the cost of loss of cellular plasticity and regenerative capacities in older parts of the colony.

## Additional files

Additional file 1:
**Phylogenetic analysis of select interstitial stem cell and germline genes in** ***Nanomia bijuga*** **.** Maximum likelihood trees are shown: (A) *vasa-1*, *vasa-2* and *pl10*, (B) *piwi* and (C) *nanos-1 and nanos-2.*
Additional file 2:
**Expression pattern of** ***vasa-1.*** Sense controls are labeled within the figure. Anterior regions or distal regions in case of zooids are up. (A,B) Nectosomal growth zone. (C,D) Siphosomal growth zone and anterior part of the siphosome. (E) Mature gastrozooid with expression in the tentacle base and forming tentilla. (F) Close-up of tentacle base shown in E. (G) Close-up of palpacle base. (H) Mature palpon. (I) Mature gastrozooid. (J) Mature female gonodendron. (K) Male gonodendron with four gonophores in different developmental stages. (L) Young female gonodendron. (M) Young male gonophore. b: bract; ba: basigaster; fg: female gonodendron; g: gastrozooid; h: horn of the growth zone; mgo: male gonophore; ne: nectophore; nst: nectosomal stem; p: palpon; pba: palpacle base; pn: pneumatophore; sst: siphosomal stem; tba: tentacle base. Additional file 3:
**Expression pattern of** ***pl10.*** Sense controls are labeled within the figure. Anterior regions or distal regions in case of zooids are up. (A,B) Nectosomal growth zone. (C) Siphosomal growth zone. (D) Siphosomal growth zone and anterior part of the siphosome. (E) Siphosomal growth zone with horn. (F) Anterior part of the siphosome. (G,H) Mature gastrozooid. (I,J) Mature palpon. (K,L) Mature female gonodendron. (M,N) Male gonodendron. ba: basigaster; fg: female gonodendron; g: gastrozooid; h: horn of the growth zone; mgo: male gonophore; ne: nectophore; nst: nectosomal stem; p: palpon; pba: palpacle base; pn: pneumatophore; sst: siphosomal stem; tba: tentacle base. Additional file 4:
**Expression pattern of** ***piwi.*** Sense controls are labeled within the figure. Anterior regions or distal regions in case of zooids are up unless stated otherwise. (A,B) Nectosomal growth zone. (C) Siphosomal growth zone. (D) Siphosomal growth zone and anterior part of the siphosome. (E) Siphosomal growth zone and anterior part of the siphosome. (F) Subsequent siphosomal fragment. *piwi* expression was found in the basigaster region of the gastrozooid at the top but not in the gastrozooid more posteriorly. (G,H) Mature gastrozooid. (I,J) Mature palpon. (K) Close-up of palpacle base shown in I. (L) Close-up of palpacle base shown in J. (M) Mature female gonodendron. (N) Mature male gonodendron. (O) Male gonophore and young female gonodendron. Lateral view of the stem. Dorsal is up. b: bract; ba: basigaster; fg: female gonodendron; g: gastrozooid; h: horn of the growth zone; mgo: male gonophore; ne: nectophore; nst: nectosomal stem; p: palpon; pba: palpacle base; pn: pneumatophore; sst: siphosomal stem; tba: tentacle base; te: tentillum. Additional file 5:
**Expression pattern of** ***nanos-1.*** Sense controls are labeled within the figure. Anterior regions or distal regions in case of zooids are up unless stated otherwise. (A,B) Nectosomal growth zone. Some unspecific signal was observed within the pneumatophore of the sense control. (C) Siphosomal growth zone. (D) Siphosomal growth zone and anterior part of the siphosome. (E) Siphosomal growth zone and anterior part of the siphosome. (F,G) Mature gastrozooid. (H,I) Mature palpon. (J,K) Mature female gonodendron. (L) Male gonodendron. (M) Male gonophores. Lateral view of the stem. ba: basigaster; fg: female gonodendron; g: gastrozooid; h: horn of the growth zone; mgo: male gonophore; ne: nectophore; nst: nectosomal stem; p: palpon; pba: palpacle base; pn: pneumatophore; sst: siphosomal stem; tba: tentacle base. Additional file 6:
**Expression of** ***nanos-2*** **stained blue** ***.*** Sense negative controls (B,D,I,L,M) are labeled within the figure. Anterior regions or distal regions in case of zooids are up. (A,B) Nectosomal growth zone. (C,D) Siphosomal growth zone. (E,F) Anterior part of the siphosome. (G) Mature cormidium with mature female and male gonophores. (H) Gastrozooid. (I) Close-up of tentacle base shown in H. (J) Proximal end of a palpon with palpacle base. (K) Tentacle base. (L) Palpacle base. (M) Cell cluster with *nanos-2* expression at the site of gonodendron formation at the base of a palpon. (N) Developing bean-shaped female gonodendron. (O) Developing female gonodendron starting to spiral. (P) Mature female gonodendron. (Q) Male gonodendron with three gonophores. (R) Mature female gonodendron. (S) Male gonophores. ba: basigaster; fg: female gonodendron; g: gastrozooid; goc: gonodendron cell cluster; h: horn of the growth zone; mgo: male gonophore; ne: nectophore; nst: nectosomal stem; p: palpon; pba: palpacle base; pn: pneumatophore; sst: siphosomal stem; tba: tentacle base.

## Abbreviations

i-cell: interstitial stem cell
EdU: 5-ethynyl-2-deoxyuridine
FSW: filtered sea water
ROV: remotely operated vehicle
TEM: transmission electron microscopy
