# Supplementary figures and images for: Stem cells in Nanomia bijuga (Siphonophora), a colonial animal with localized growth zones
Source: EvoDevo. 2015 May 27;6:22. doi: 10.1186/s13227-015-0018-2 (PMC4471933; doi:10.1186/s13227-015-0018-2)

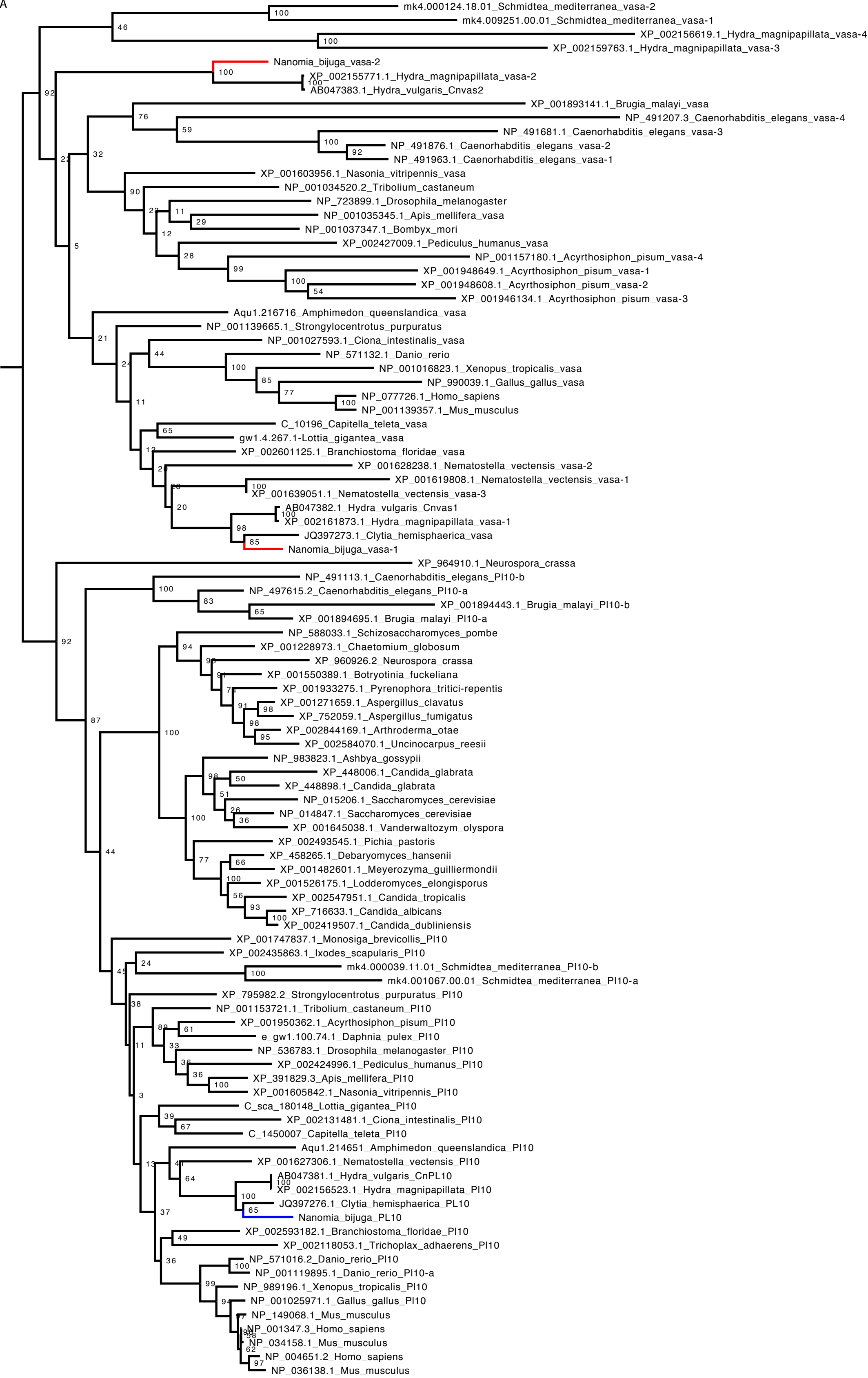

B

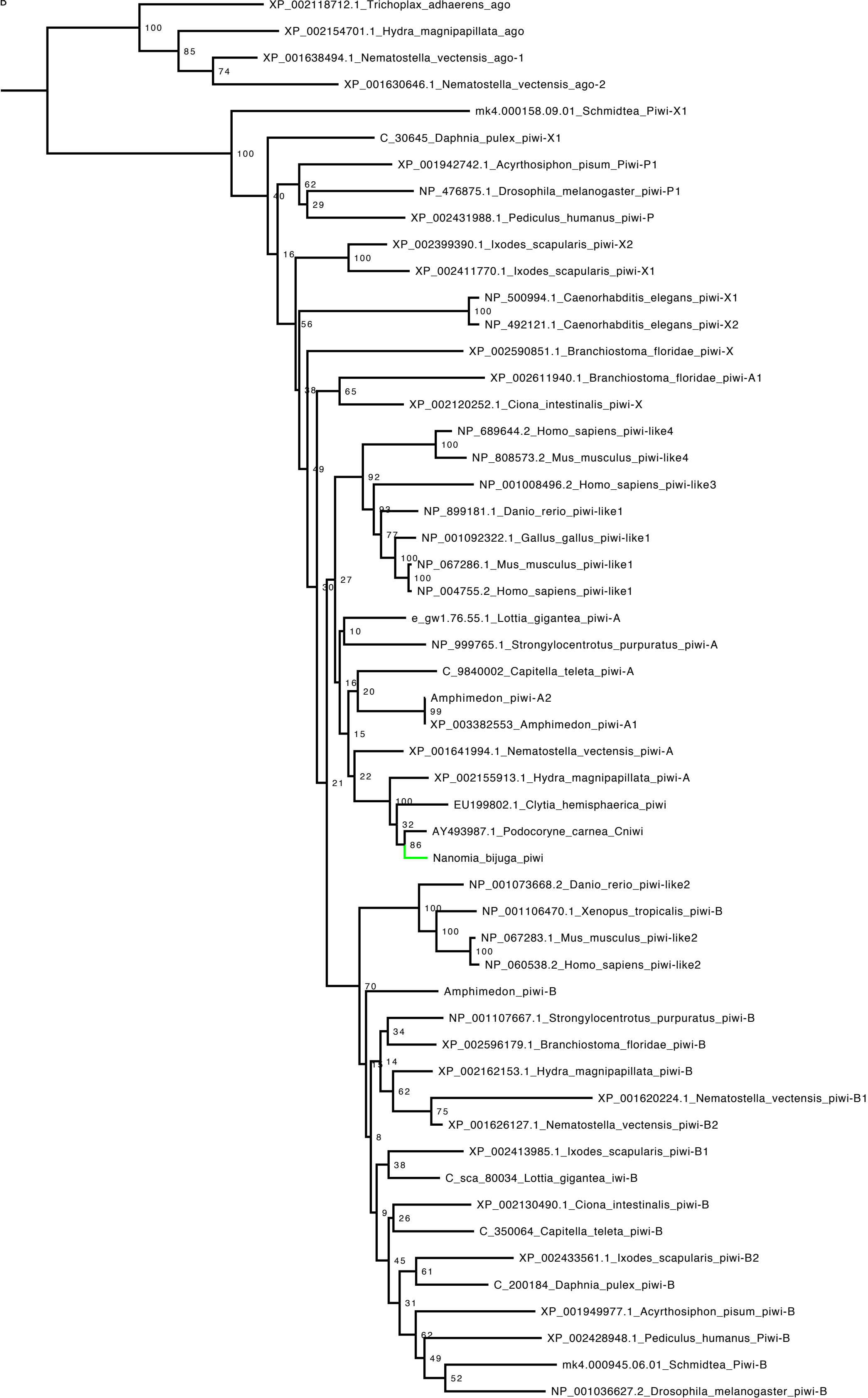

0.6

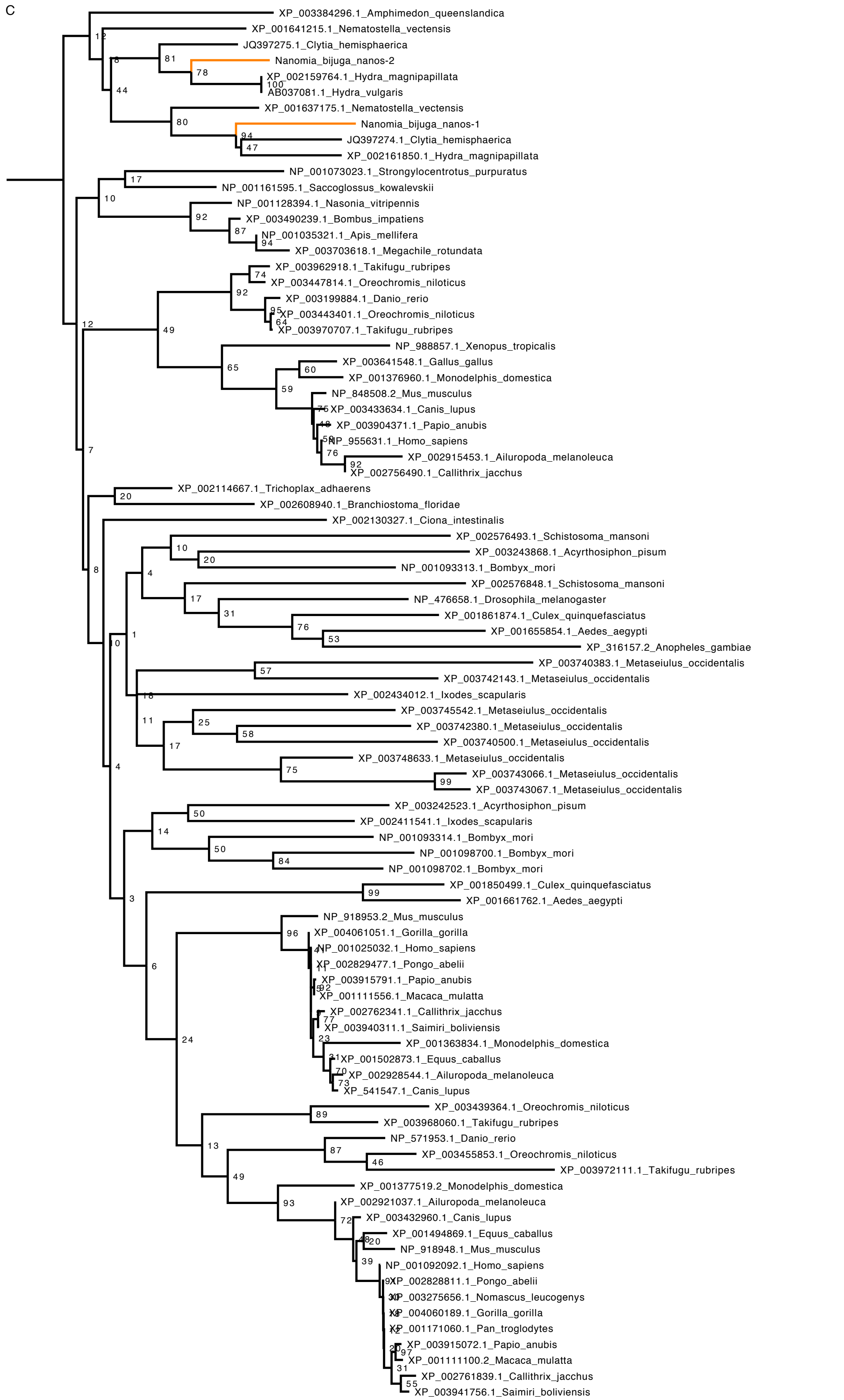

Supplement: Additional file 1: — Phylogenetic analysis of select interstitial stem cell and germline genes in Nanomia bijuga . Maximum likelihood trees are shown: (A) vasa-1, vasa-2 and pl10, (B) piwi and (C) nanos-1 and nanos-2. [file 13227_2015_18_MOESM1_ESM.pdf]

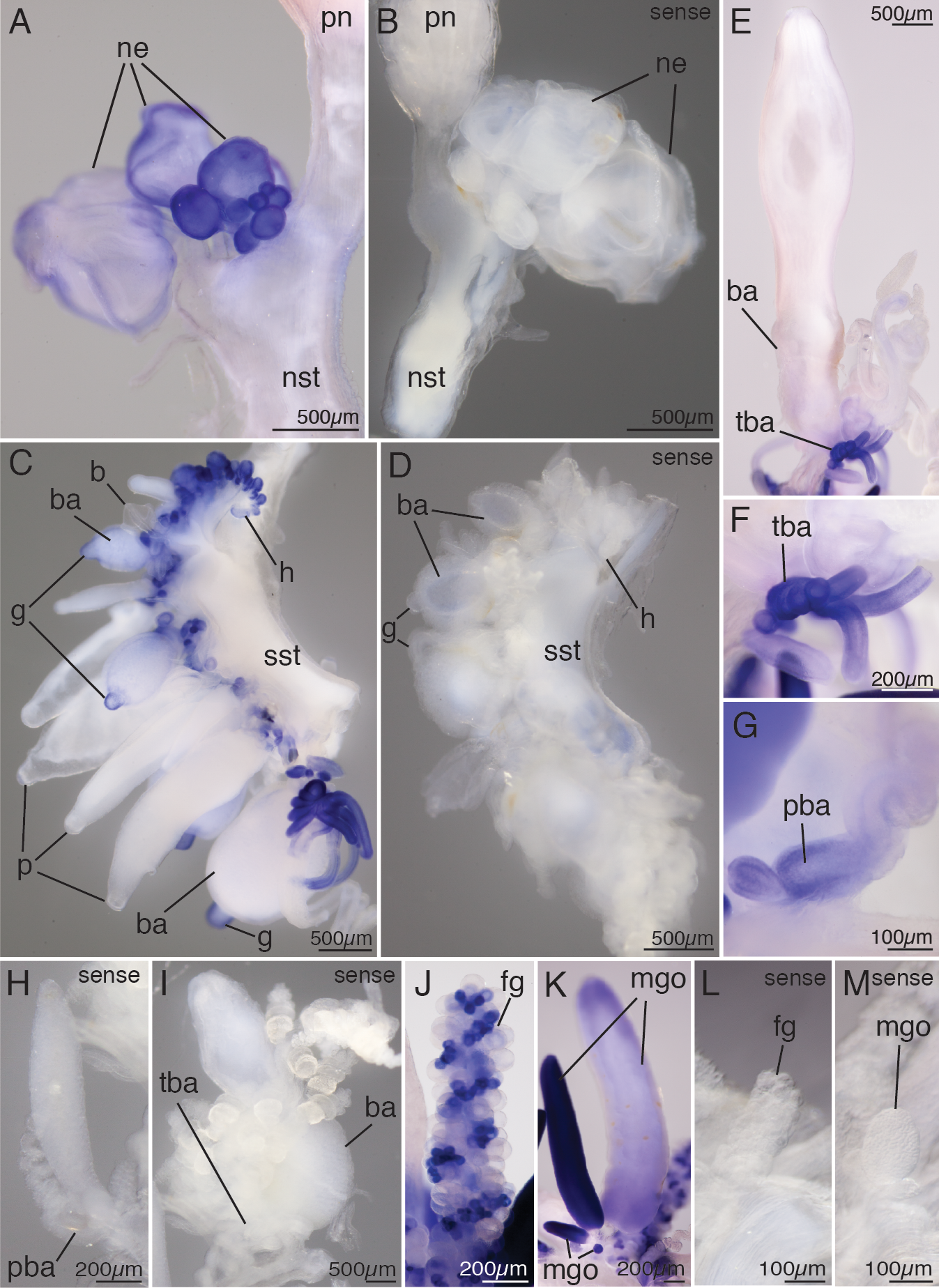

Supplement: Additional file 2: — Expression pattern of vasa-1. Sense controls are labeled within the figure. Anterior regions or distal regions in case of zooids are up. (A,B) Nectosomal growth zone. (C,D) Siphosomal growth zone and anterior part of the siphosome. (E) Mature gastrozooid with expression in the tentacle base and forming tentilla. (F) Close-up of tentacle base shown in E. (G) Close-up of palpacle base. (H) Mature palpon. (I) Mature gastrozooid. (J) Mature female gonodendron. (K) Male gonodendron with four gonophores in different developmental stages. (L) Young female gonodendron. (M) Young male gonophore. b: bract; ba: basigaster; fg: female gonodendron; g: gastrozooid; h: horn of the growth zone; mgo: male gonophore; ne: nectophore; nst: nectosomal stem; p: palpon; pba: palpacle base; pn: pneumatophore; sst: siphosomal stem; tba: tentacle base. [file 13227_2015_18_MOESM2_ESM.png]

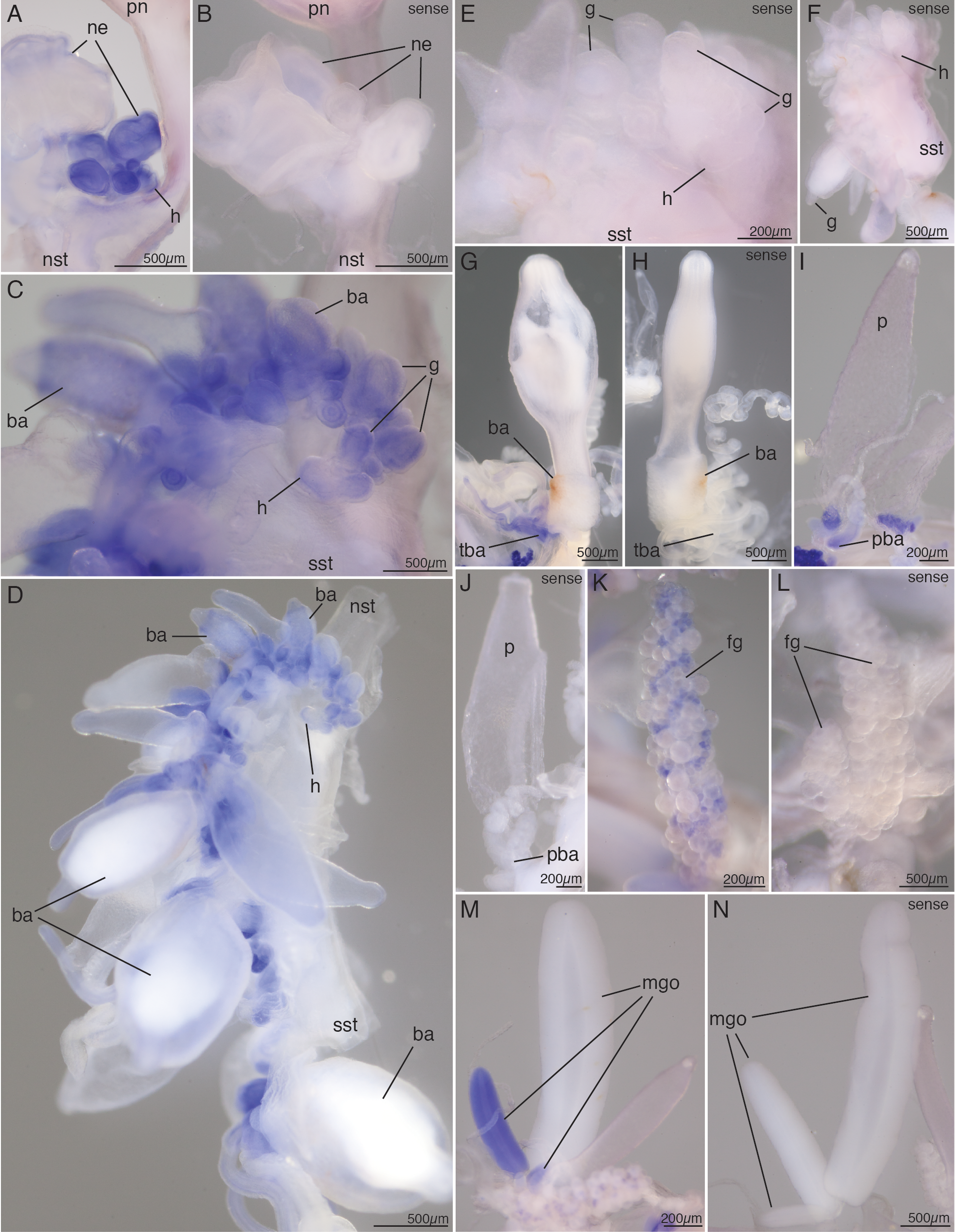

Supplement: Additional file 3: — Expression pattern of pl10. Sense controls are labeled within the figure. Anterior regions or distal regions in case of zooids are up. (A,B) Nectosomal growth zone. (C) Siphosomal growth zone. (D) Siphosomal growth zone and anterior part of the siphosome. (E) Siphosomal growth zone with horn. (F) Anterior part of the siphosome. (G,H) Mature gastrozooid. (I,J) Mature palpon. (K,L) Mature female gonodendron. (M,N) Male gonodendron. ba: basigaster; fg: female gonodendron; g: gastrozooid; h: horn of the growth zone; mgo: male gonophore; ne: nectophore; nst: nectosomal stem; p: palpon; pba: palpacle base; pn: pneumatophore; sst: siphosomal stem; tba: tentacle base. [file 13227_2015_18_MOESM3_ESM.png]

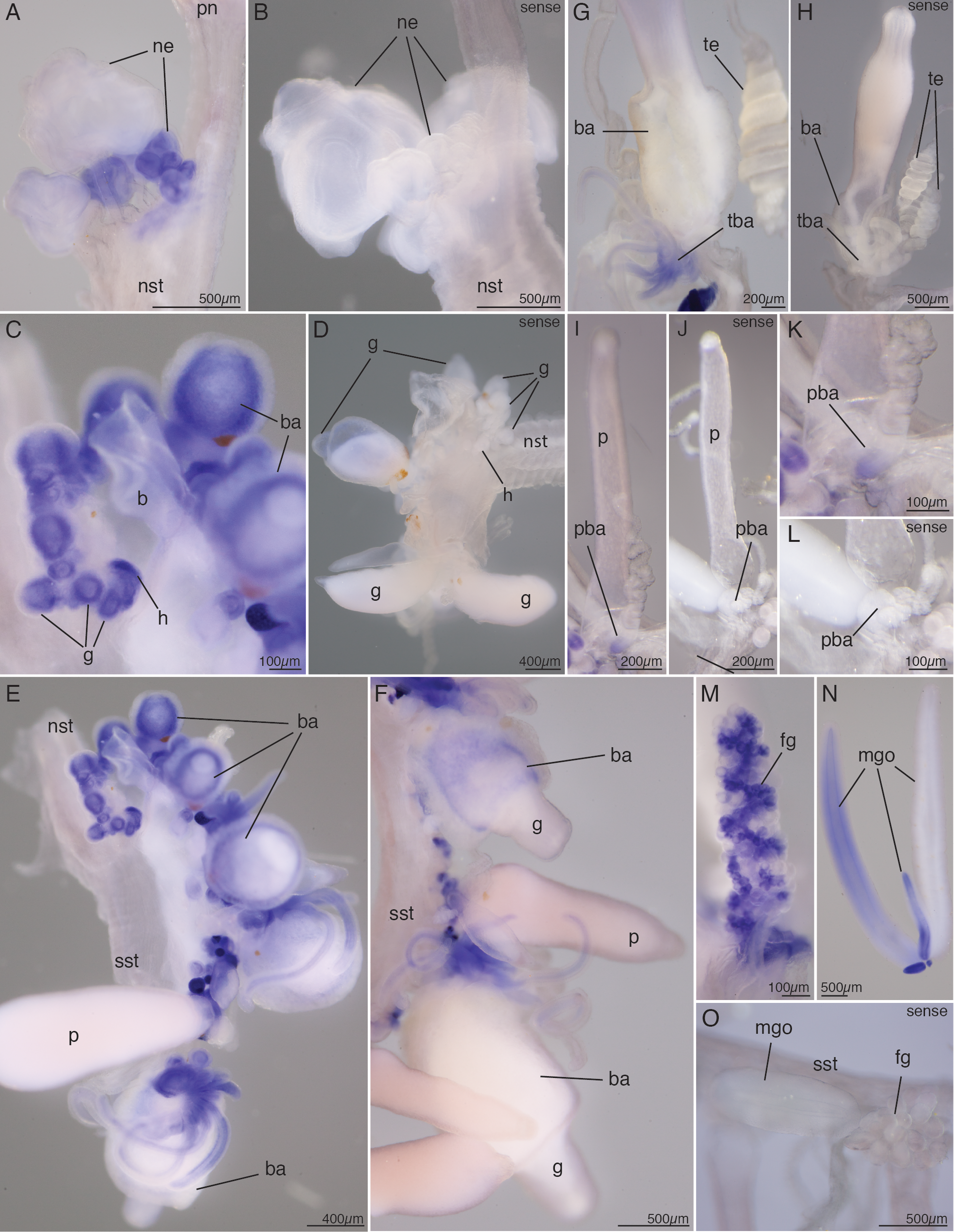

Supplement: Additional file 4: — Expression pattern of piwi. Sense controls are labeled within the figure. Anterior regions or distal regions in case of zooids are up unless stated otherwise. (A,B) Nectosomal growth zone. (C) Siphosomal growth zone. (D) Siphosomal growth zone and anterior part of the siphosome. (E) Siphosomal growth zone and anterior part of the siphosome. (F) Subsequent siphosomal fragment. piwi expression was found in the basigaster region of the gastrozooid at the top but not in the gastrozooid more posteriorly. (G,H) Mature gastrozooid. (I,J) Mature palpon. (K) Close-up of palpacle base shown in I. (L) Close-up of palpacle base shown in J. (M) Mature female gonodendron. (N) Mature male gonodendron. (O) Male gonophore and young female gonodendron. Lateral view of the stem. Dorsal is up. b: bract; ba: basigaster; fg: female gonodendron; g: gastrozooid; h: horn of the growth zone; mgo: male gonophore; ne: nectophore; nst: nectosomal stem; p: palpon; pba: palpacle base; pn: pneumatophore; sst: siphosomal stem; tba: tentacle base; te: tentillum. [file 13227_2015_18_MOESM4_ESM.png]

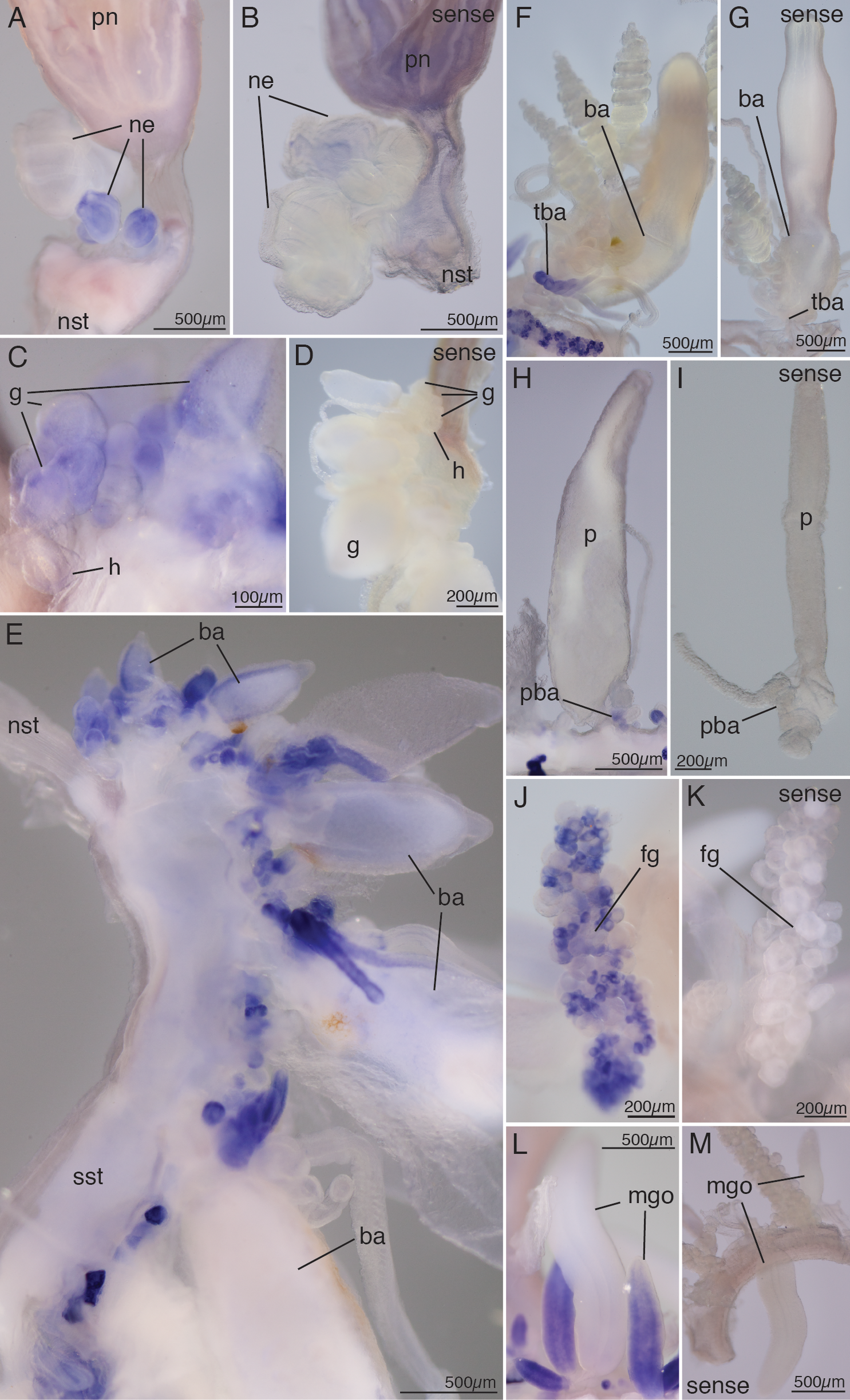

Supplement: Additional file 5: — Expression pattern of nanos-1. Sense controls are labeled within the figure. Anterior regions or distal regions in case of zooids are up unless stated otherwise. (A,B) Nectosomal growth zone. Some unspecific signal was observed within the pneumatophore of the sense control. (C) Siphosomal growth zone. (D) Siphosomal growth zone and anterior part of the siphosome. (E) Siphosomal growth zone and anterior part of the siphosome. (F,G) Mature gastrozooid. (H,I) Mature palpon. (J,K) Mature female gonodendron. (L) Male gonodendron. (M) Male gonophores. Lateral view of the stem. ba: basigaster; fg: female gonodendron; g: gastrozooid; h: horn of the growth zone; mgo: male gonophore; ne: nectophore; nst: nectosomal stem; p: palpon; pba: palpacle base; pn: pneumatophore; sst: siphosomal stem; tba: tentacle base. [file 13227_2015_18_MOESM5_ESM.png]

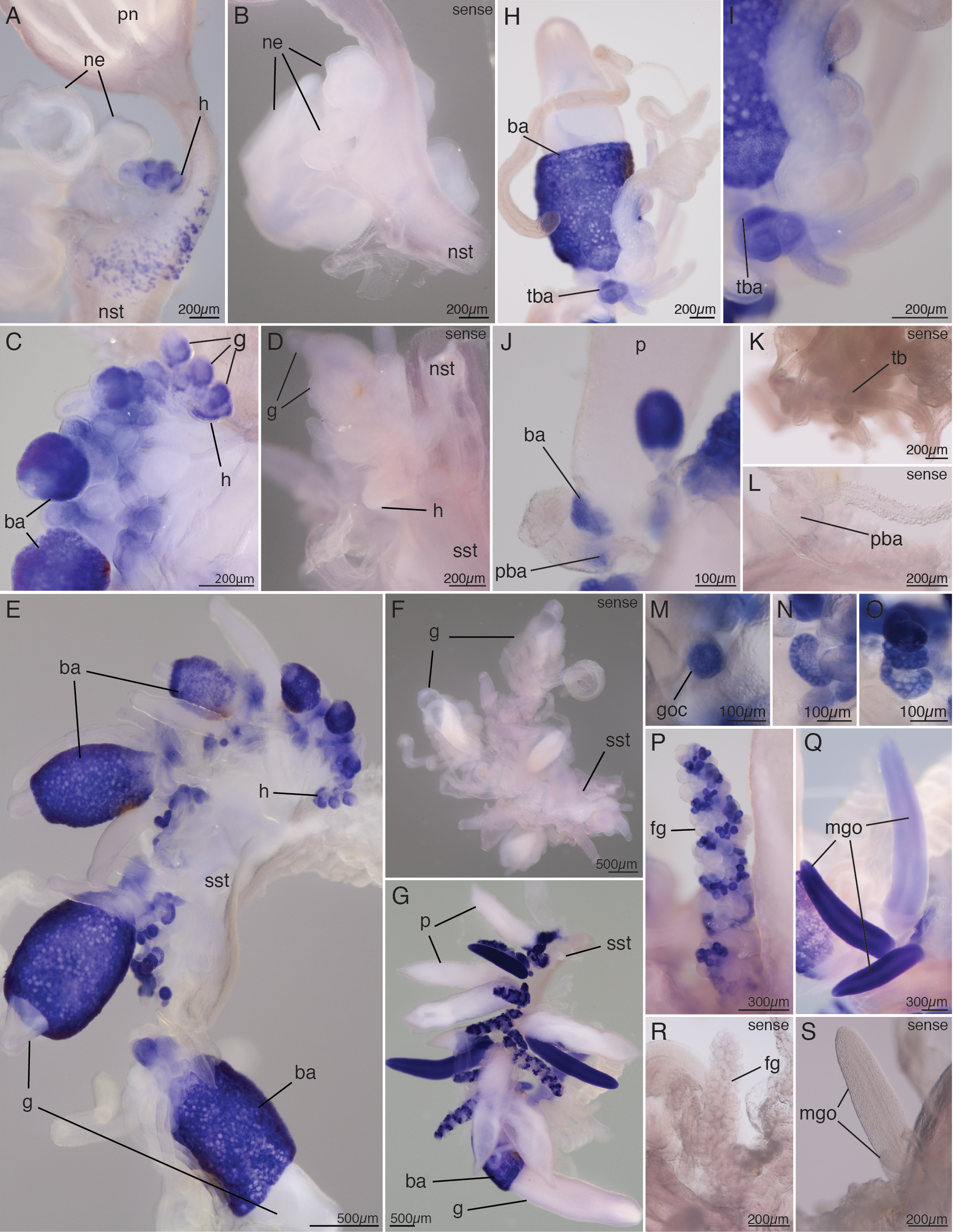

Supplement: Additional file 6: — Expression of nanos-2 stained blue . Sense negative controls (B,D,I,L,M) are labeled within the figure. Anterior regions or distal regions in case of zooids are up. (A,B) Nectosomal growth zone. (C,D) Siphosomal growth zone. (E,F) Anterior part of the siphosome. (G) Mature cormidium with mature female and male gonophores. (H) Gastrozooid. (I) Close-up of tentacle base shown in H. (J) Proximal end of a palpon with palpacle base. (K) Tentacle base. (L) Palpacle base. (M) Cell cluster with nanos-2 expression at the site of gonodendron formation at the base of a palpon. (N) Developing bean-shaped female gonodendron. (O) Developing female gonodendron starting to spiral. (P) Mature female gonodendron. (Q) Male gonodendron with three gonophores. (R) Mature female gonodendron. (S) Male gonophores. ba: basigaster; fg: female gonodendron; g: gastrozooid; goc: gonodendron cell cluster; h: horn of the growth zone; mgo: male gonophore; ne: nectophore; nst: nectosomal stem; p: palpon; pba: palpacle base; pn: pneumatophore; sst: siphosomal stem; tba: tentacle base. [file 13227_2015_18_MOESM6_ESM.png]
